# Structural Differences Between Healthy Subjects and Patients With Schizophrenia or Schizoaffective Disorder: A Graph and Control Theoretical Perspective

**DOI:** 10.3389/fpsyt.2021.669783

**Published:** 2021-06-28

**Authors:** Cristiana Dimulescu, Serdar Gareayaghi, Fabian Kamp, Sophie Fromm, Klaus Obermayer, Christoph Metzner

**Affiliations:** ^1^Neural Information Processing, Institute of Software Engineering and Theoretical Computer Science, Technische Universität Berlin, Berlin, Germany; ^2^Bernstein Center for Computational Neuroscience Berlin, Berlin, Germany; ^3^Department of Psychiatry and Psychotherapy, Charité Campus Mitte, Charité—Universitätsmedizin Berlin, Berlin, Germany; ^4^Biocomputation Group, School of Physics, Engineering and Computer Science, University of Hertfordshire, Hatfield, United Kingdom

**Keywords:** schizophrenia, connectome, graph theory, control theory, dysconnectivity

## Abstract

The coordinated dynamic interactions of large-scale brain circuits and networks have been associated with cognitive functions and behavior. Recent advances in network neuroscience have suggested that the anatomical organization of such networks puts fundamental constraints on the dynamical landscape of brain activity, i.e., the different states, or patterns of regional activation, and transition between states the brain can display. Specifically, it has been shown that densely connected, central regions control the transition between states that are “easily” reachable (in terms of expended energy), whereas weakly connected areas control transitions to states that are hard-to-reach. Changes in large-scale brain activity have been hypothesized to underlie many neurological and psychiatric disorders. Evidence has emerged that large-scale dysconnectivity might play a crucial role in the pathophysiology of schizophrenia, especially regarding cognitive symptoms. Therefore, an analysis of graph and control theoretic measures of large-scale brain connectivity in patients offers to give insight into the emergence of cognitive disturbances in the disorder. To investigate these potential differences between patients with schizophrenia (SCZ), patients with schizoaffective disorder (SCZaff) and matched healthy controls (HC), we used structural MRI data to assess the microstructural organization of white matter. We first calculate seven graph measures of integration, segregation, centrality and resilience and test for group differences. Second, we extend our analysis beyond these traditional measures and employ a simplified noise-free linear discrete-time and time-invariant network model to calculate two complementary measures of controllability. Average controllability, which identifies brain areas that can guide brain activity into different, easily reachable states with little input energy and modal controllability, which characterizes regions that can push the brain into difficult-to-reach states, i.e., states that require substantial input energy. We identified differences in standard network and controllability measures for both patient groups compared to HCs. We found a strong reduction of betweenness centrality for both patient groups and a strong reduction in average controllability for the SCZ group again in comparison to the HC group. Our findings of network level deficits might help to explain the many cognitive deficits associated with these disorders.

## 1. Introduction

Ample evidence has emerged that dysconnectivity, i.e., network-level abnormalities in the connectivity between brain regions, might play a central role in the pathophysiology of schizophrenia (1–4). While traditionally analysis of structural connectivity has looked at gray and white matter volume and tissue anisotropy, over the last decades, network neuroscience, interpreting the brain as a complex network of interconnected regions, has emerged and applied graph and network science to understand structure-function relationships in the brain (5). Therefore, a graph-theoretical analysis of brain networks is well-suited to address the network-level abnormalities that according to the dysconnectivity hypothesis might lie at the heart of the disorder. Graph theoretical measures allow for a characterization of changes to the topology of brain networks that goes beyond a description of in- or decreased connectivity between regions. Overall, studies applying network science techniques have identified disturbances regarding integration and segregation properties in structural and functional networks in schizophrenia (6–8) as well as reduced connectivity within the central hub structure, i.e., a set of densely connected regions that provides links between functional subnetworks (9). Therefore, we hypothesized that patients with schizophrenia and schizoaffective disorder would show reductions in integration, segregation and centrality measures. Moving beyond a graph theoretical perspective, Gu et al. (10) employed a control-theoretic framework to gain a deeper understanding of the dynamic interactions between large-scale brain networks and their relation to cognitive abilities. This framework sees the brain as a dynamic network that shows a repertoire of potential brain states, where a brain state can simply be viewed as a distinct spatio-temporal pattern of brain activity, that is continually revisited against a background of noisy neural activity (11, 12). The dynamics of this state revisiting is thought to underlie cognition (13, 14), reflect overall capacity (15) and are likely related to changing mental states. Furthermore, since cognitive deficits constitute a core feature of schizophrenia (16) it seems likely that they are related to changes in the dynamic transitions between brain states. Gu et al. (10) now demonstrated that the anatomical structure constrains this temporal evolution of activity and dynamic changes of brain states. Therefore, control theoretic results can be harnessed to derive insights into the effect of structural features on brain dynamics and into how they constrain the transition between brain states. Their results imply that densely connected areas (e.g., in the default mode network) enable the movement of brain activity to many easily reachable states, i.e., state transitions that require the least amount of energy, whereas weakly connected regions (e.g., in the cognitive control systems) help the brain to transition to states that are hard to reach, again in terms of energy. Importantly, the results of Gu et al. (10) highlight that changes to large-scale brain dynamics can, at least to some degree, be predicted from controllability measures calculated from the structural, anatomical connectivity alone. This suggests that the global, large-scale activity changes in schizophrenia, which have been associated with deficits in cognitive processes, might be reflected in control theoretic measures of structural connectivity. Therefore, we hypothesized that patients might display disturbances in measures of network controllability. Since the affective symptom components of patients with schizoaffective disorder are more similar to patients with bipolar disorder than to patients with schizophrenia and since difference in the central hub structure between patients with schizophrenia and bipolar disorder have been identified (17), we further hypothesized that patients with schizoaffective disorder and patients with schizophrenia might show differences in network and control measures, especially in centrality measures.

To address this hypothesis, we built structural brain networks from DTI and T1w MRI using a freely available data set from the Center of Biomedical Research Excellence (COBRE) data repository of patients with schizophrenia and schizoaffective disorder and subsequently performed a graph- and control-theoretic analysis. We investigated graph measures of integration, segregation, centrality and resilience together with two measures of controllability, average, and modal controllability. We found a strong difference between healthy controls and patients for betweenness centrality and, importantly, strong differences in controllability between the groups.

## 2. Methods

### 2.1. Data Set

We queried the SchizConnect database (http://schizconnect.org) SchizConnect to obtain our study sample. We used the following query “study: COBRE, subject: schizophrenia_broad, symptoms_psychpathology: PANSS protocol: structural” to obtain the data for the patient groups. This resulted in 83 SCZ and 11 SCZaff subjects. A second SchizConnect query “study: COBRE, subject: no_known_disorder, protocol: structural” was used to obtain data for 91 healthy control subjects. We did not query other studies on SchizConnect to avoid artifacts introduced by different imaging sites. After preprocessing we excluded subjects where head movement and eddy current correction or the brain extraction (BET) was not successful on the T1w images or where fitting the diffusion model and probabilistically tracking of fibres failed on the diffusion imaging data. This resulted in 43 SCZ and 9 SCZaff subjects. We then randomly chose 43 healthy control subjects for which (a) preprocessing was successful and b) which did not significantly differ from the two patient groups with respect to age and gender distribution. Because of the relatively high rejection rate for the schizophrenia patient group, we wanted to verify that the resulting final sample was still representative of the total sample and thus compared the total sample (83 subjects) with the final sample (43 subjects). Specifically, we performed *t*-tests to test for statistically significant differences in age (*t* = 0.48, *p* = 0.63), illness duration (*t* = 0.36, *p* = 0.72), PANSS positive (*t = -0.11, p = 0.91*), PANSS negative (*t* = 0.13, *p* = 0.89), PANSS general (*t = 0.32, p* = 0.75), PANSS total (*t* = 0.19, *p* = 0.85), and a chi-square test to test for differences in gender (χ^2^ = 1.63, *p* = 0.20). We performed the same analysis for the full SCZaff sample (11 subjects) and the final SCZaff sample (9 subjects): age (*t = -0.25, p* = 0.81), illness duration (*t* = −0.57, *p* = 0.57), PANSS positive (*t = -0.64, p = 0.53*), PANSS negative (*t = -0.37, p* = 0.71), PANSS general (*t = -0.09, p* = 0.93), PANSS total (*t* = −0.31, *p* = 0.76), and (χ^2^ = −0.07, *p* = 0.80). Neither of the measures differed significantly between the initial full patient sample and the final patient sample.

We thus obtained a final sample of 43 healthy control subjects, 43 patients with schizophrenia and 9 patients with schizoaffective disorder. Patients received antipsychotic medication. Symptom severity in patients was assessed using the Positive and Negative Syndrome Scale (PANSS) (18). Written informed consent was obtained from all participants, and the study was approved by the regional ethics committee. The groups did not differ significantly in terms of age and gender, and the patient groups also did not differ significantly in terms of symptoms as measured with PANSS, duration of illness and medication dosage as measured with the chlorpromazine-equivalent (CPZ-equivalent) dosage (see Table 1). Patients showed no change in symptomatology or type/dose of antipsychotic medication during the 3 months before the assessment [for more details see (19)].

**Table 1:** Demographics and clinical characteristics.

	**HC**	**SCZ**	**SCZaff**	**Statistics, p-value**
Group size	43	43	9	
Age	36.70 (11.04)	38.72 (14.02)	39.00 (12.60)	HC/SCZ: *t* = −0.73, *p* = 0.46
(years)				HC/SCZaff: *t* = −0.54, *p* = 0.59
Gender	11/32	12/31	2/7	HC/SCZ: χ^2^ = 0.06, *p* = 0.81
(female/male)				HC/SCZaff: χ^2^ = 0.04, *p* = 0.83
PANSS positive	–	14.34 (4.94)	15.78 (3.12)	SCZ/SCZaff: *t* = −0.75, *p* = 0.45
PANSS negative	–	14.45 (4.90)	16.33 (4.45)	SCZ/SCZaff: *t* = −1.04, *p* = 0.30
PANSS general	–	28.47 (8.16)	33.78 (8.98)	SCZ/SCZaff: *t* = −1.65, *p* = 0.10
PANSS total	–	57.53 (13.44)	65.89 (14.93)	SCZ/SCZaff: *t* = −1.63, *p* = 0.11
CPZ equiv. dose	–	373.54 (335.70)	375.0 (210.82)	SCZ/SCZaff: *t* = −0.01, *p* = 0.99
Illness duration	–	16.89 (12.99)	16.10 (12.12)	SCZ/SCZaff: *t* = −0.84, *p* = 0.41

*Data are shown as mean(standard deviation). Age, medication and illness duration differences between groups were compared using an independent samples t-test and differences in gender distribution using a chi-square test*.

Data collection was performed using a Siemens Magnetom Trio 3T MR scanner. Structural images (high resolution T1-weighted) were acquired using a five-echo MPRAGE sequence with the following parameters: repetition time (TR) = 2,530 ms; echo time (TE) = 1.64, 3.5, 5.36, 7.22, 9.08 ms; inversion time (TI) = 1,200 ms; flip angle (FA) = 7°; field of view (FOV) = 256 × 256 mm; matrix = 256 × 256; slice thickness = 1 mm; 192 sagittal slices. Diffusion tensor imaging (DTI) data were acquired using a single-shot EPI sequence with TR/TE = 9,000/84 ms; FA = 90°; FOV =256 mm × 256 mm; matrix = 128 × 128; slice thickness = 2 mm without gap; 72 axial slices; 30 non-collinear diffusion gradients (b = 800 s/mm^2^) and 5 non-diffusion-weighted images (b = 0 s/mm^2^) equally interspersed between the 30 gradient directions. For more information see also (20).

### 2.2. Data Preprocessing and Tractography

We conducted preprocessing of anatomical and diffusion images using a semi-automatic pipeline implemented in the FSL toolbox (www.fmrib.ox.ac.uk/fsl, FMRIB, Oxford). For the anatomical images, preprocessing included removal of non-brain tissue and brain extraction using the brain extraction toolbox (BET) implemented in FSL, as well as the generation of a brain mask. After extraction, images were checked for quality and subsequently, 94 cortical and subcortical regions were defined according to the automated anatomical atlas (AAL2) described in (21).

For diffusion data, we performed brain extraction on the b0 images using BET. After correcting the data for head movement and eddy current distortions, we fitted a probabilistic diffusion model to the data by using the Bayesian Estimation of Diffusion Parameters Obtained using Sampling Techniques (BEDPOSTX) FSL toolbox. Finally, we linearly registered each b0 image to the corresponding subject's anatomical T1 image, transformed the high-resolution anatomical mask volumes containing the cortical parcellations to the subject's diffusion space, and ran probabilistic tractography with 5,000 samples per voxel using FSL's PROBTRACKX algorithm (22).

### 2.3. Network Construction

We defined the weight of a connection between two regions *i* and *j* to be the inverse of the normalized number of fibres *nf*_*ij*_ determined through the probabilistic tracking:

(1) $$\begin{array}{l} w_{ij}=\frac{1}{nf_{ij}}, \end{array}$$

if *nf*_*ij*_ > *T*, with a certain threshold *T*. If not mentioned otherwise, we used a threshold of *T* = 0.Using this definition, the larger the weight between two regions the weaker the connectivity is between these regions, or in other words, the higher cost is to go from one node to the other. This approach is similar to previously used definitions of weighted structural networks (23–25).

### 2.4. Network Measures

We followed Rubinov and Sporns (26) and defined the network measures as follows. Let *W* = *w*_*ij*_ be the weighted matrix describing the pair-wise connectivity between regions constructed from the DTI scans. Let *N* be the set of nodes in the network with *n*: = |*N*| = 94. Then we can define some basic measures describing a network. First, the weighted degree of node *i* can be defined as

(2) $$\begin{array}{l} k_{i}^{w}=\underset{j\in N}{\sum }w_{ij}. \end{array}$$

The shortest weighted path length between two nodes *i* and *j* is given by

(3) $$\begin{array}{l} d_{ij}^{w}=\underset{a_{uv}\in g_{i-j}^{w}}{\sum }f\left(w_{uv}\right), \end{array}$$

where f is the inverse of the weight and $g_{i-j}^{w}$ is the shortest weighted path between *i* and *j*. Lastly, the weighted geometric mean of triangles around a node *i* can be calculated as

(4) $$\begin{array}{l} t_{i}^{w}=\frac{1}{2}\underset{j,h\in N}{\sum }{\left(w_{ij}w_{ih}w_{jh}\right)}^{1/3}. \end{array}$$

With the weighted degree, the shortest weighted path length and the weighted geometric mean of triangles, we have the basic measures that will allow us to define our measures of integration, segregation, and centrality. While measures of integration are often based on the path length, segregation measures are typically based on triangle counts, with triangles being the simplest fully connected structure with more than two nodes, i.e., the smallest cluster. Measures of centrality can be based on the degree or on the shortest paths. Resilience measures are often more complex measures based on the node degrees.

With these basic measures we can now define the network measures of interest.

#### 2.4.1. Integration

First, we define two measures of integration: characteristic path length and global efficiency. The weighted characteristic path length (for short, since we only deal with weighted graphs in this study, the characteristic path length) describes the average shortest path length between two nodes in the network, where the average is taken over all possible pairs of nodes *i* and *j*. It is defined as

(5) $$\begin{array}{l} L^{w}=\frac{1}{n}\underset{i\in N}{\sum }\frac{\sum_{i\in N,j\neq i}d_{ij}^{w}}{n-1}. \end{array}$$

The global efficiency is defined as

(6) $$\begin{array}{l} E^{w}=\frac{1}{n}\underset{i\in N}{\sum }\frac{\sum_{i\in N,j\neq i}{\left(d_{ij}^{w}\right)}^{-1}}{n-1}. \end{array}$$

#### 2.4.2. Segregation

Next, we define two measures of segregation: the weighted clustering coefficient (for short the clustering coefficient) and the weighted transitivity (for short the transitivity). The clustering coefficient can be calculated as

(7) $$\begin{array}{l} C^{w}=\frac{1}{n}\underset{i\in N}{\sum }\frac{2t_{i}^{w}}{k_{i}\left(k_{i}-1\right)} \end{array}$$

and the transitivity is given by

(8) $$\begin{array}{l} T^{w}=\frac{1}{n}\frac{\sum_{i\in N}2t_{i}^{w}}{\sum_{i\in N}k_{i}\left(k_{i}-1\right)}. \end{array}$$

#### 2.4.3. Centrality

We then introduce two measures of centrality: closeness centrality and betweenness centrality. We define closeness centrality as the average of nodal closeness centrality over all nodes, where nodal closeness centrality of node *i* is given by

(9) $$\begin{array}{l} {\left(L_{i}^{w}\right)}^{-1}=\frac{n-1}{\sum_{i\in N,j\neq i}d_{ij}^{w}} \end{array}$$

and, similarly, betweenness centrality as the average of nodal betweenness centrality over all nodes, where nodal betweenness centrality of node *i* calculated as

(10) $$\begin{array}{l} b_{i}=\frac{1}{\left(n-1\right)\left(n-2\right)}\underset{h,j\in N;h\neq j,h\neq i,j\neq i}{\sum }\frac{\rho_{hj}^{w}\left(i\right)}{\rho_{hj}^{w}}, \end{array}$$

where $\rho_{hj}^{w}$ is the number of shortest weighted paths between *h* and *j*, and $\rho_{hj}^{w}\left(i\right)$ is the number of shortest weighted paths between *h* and *j* that pass through *i*.

#### 2.4.4. Resilience

Lastly, we also defined a measure of network resilience: (weighted) assortativity. (Weighted) assortativity can be defined as

(11) $$\begin{array}{l} r^{w}=\frac{l^{-1}\sum_{\left(i,j\right)\in L}w_{ij}k_{i}^{w}k_{j}^{w}-{\left[l^{-1}\sum_{\left(i,j\right)\in L}w_{ij}\left(k_{i}^{w}+k_{j}^{w}\right)\right]}^{2}}{l^{-1}\sum_{\left(i,j\right)\in L}\frac{1}{2}w_{ij}\left({\left(k_{i}^{w}\right)}^{2}+{\left(k_{j}^{w}\right)}^{2}\right)-{\left[l^{-1}\sum_{\left(i,j\right)\in L}w_{ij}\left(k_{i}^{w}+k_{j}^{w}\right)\right]}^{2}}. \end{array}$$

### 2.5. Controllability Measures

Here we utilized the control theoretic notion of controllability which gives theoretical insight into the effect local changes of dynamics might have. We chose two different measures based on a linear control setting: *average controllability* and *modal controllability*. Average controllability is given by the average input energy to the control nodes over all possible target states. Typically, nodes with high average controllability can control networks dynamics over nearby target states in an energy efficient way. On the other hand, modal controllability describes how well a node can control all network modes. High modal controllability means that a node is able to reach all modes of a network and, thus, can force the dynamics into hard-to-reach target states.

To define the controllability measures, we assume a simplified noise-free linear discrete-time and time-invariant network model of the form

(12) $$\begin{array}{l} x\left(t+1\right)=Wx\left(t\right)+B_{k}u_{k}\left(t\right), \end{array}$$

where *x* is the state of the network, *W* is again the weighted adjacency matrix of the network, the input matrix *B*_*k*_ specifies the control nodes and *u*_*k*_ defines the control strategy over time. In our setting, we always choose a single control node *i*, which means that *B*_*i*_ is simply a unit vector with entry at row *i*. This allows us to define the so-called controllability Gramian as

(13) $$\begin{array}{l} G_{i}=\overset{\infty }{\underset{\tau =0}{\sum }}W^{\tau }B_{i}B_{i}^{T}W^{\tau }. \end{array}$$

The controllability Gramian contains information on the controllability of the network under the specific control *B*_*i*_. In fact, one classic results from control theory states that the network is controllable from the node *i* if and only if the controllability Gramian is invertible. We then define average controllability of node *i* simply as the trace *Tr*(*G*_*i*_) of the controllability Gramian [see (10, 27)]. Average controllability here can be interpreted as the average input energy from the control node over all possible network states (28). regions with high average controllability can be interpreted as the most influential nodes since they, on average, need the least energy to steer the dynamical system (10). The average controllability of the network is then defined as the mean of the average controllability over all nodes in the network. Modal controllability of node *i* will be defined as

(14) $$\begin{array}{l} \phi_{i}=\overset{n}{\underset{j=1}{\sum }}\left(1-\lambda_{j}^{2}\left(W\right)\right)v_{ij} \end{array}$$

where *V* = (*v*_*ij*_) is the matrix of eigenvectors from the weighted adjacency matrix *W* and λ_*j*_(*W*) are the eigenvalues of *W*. Modal controllability provides a scaled measure of the controllability of the modes of a brain region. Regions with high modal controllability can control all modes of a dynamic network and therefore drive its dynamics into difficult-to-reach configurations (29).

## 3. Results

### 3.1. Graph Measures

We first compared seven standard graph measures in the two patient groups against the shared control of healthy subjects. The graph measures were: characteristic path length and global efficiency (two measures of integration), cluster coefficient and transitivity (two measures of segregation), betweenness and closeness centrality (two measures of centrality), and assortativity (a measure of resilience).

#### 3.1.1. Integration

**Figure 1:**
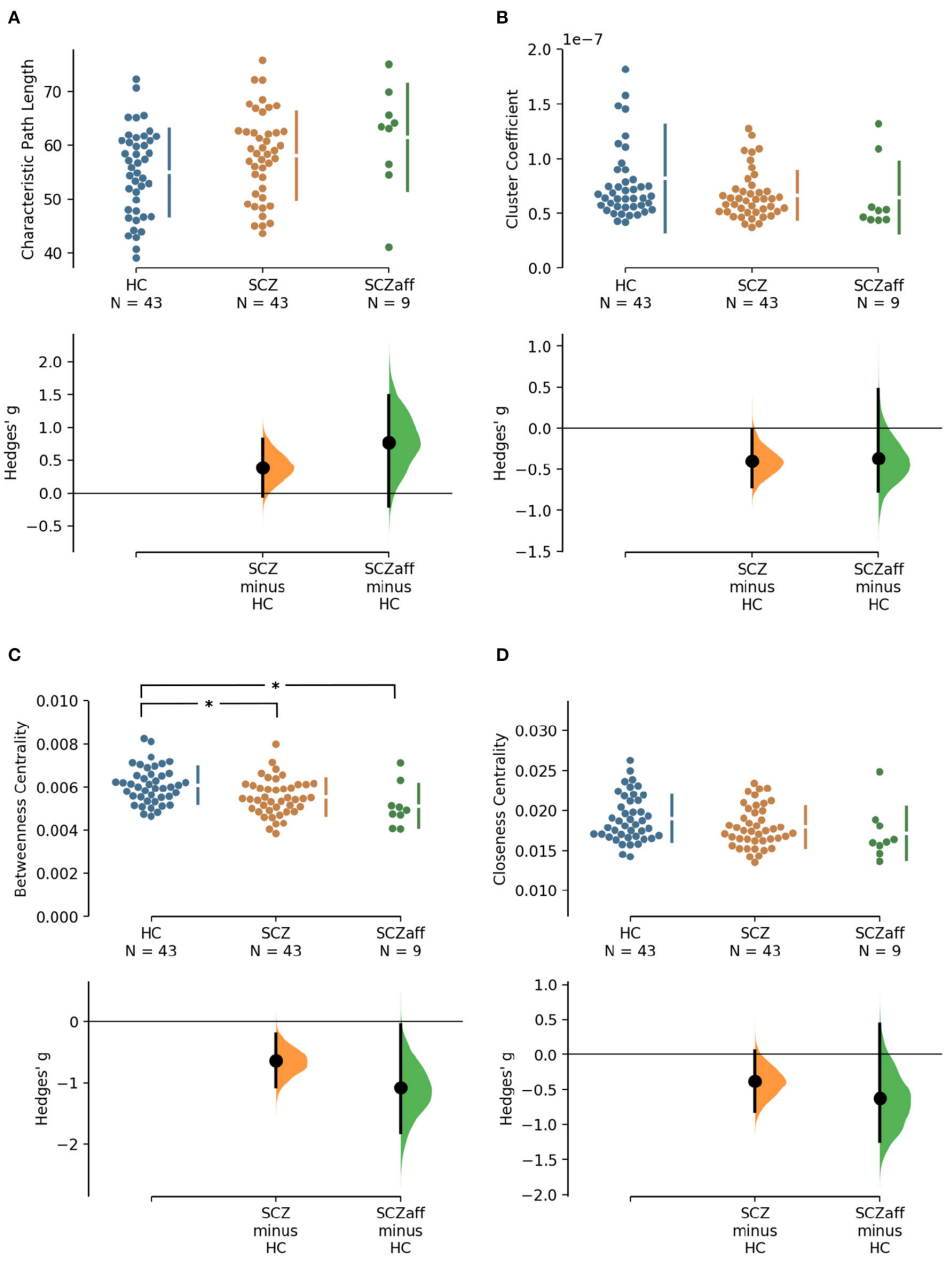
Hedge's *g* for the comparisons of various graph measures for the SCZ and SCZaff groups against the HC group are shown in the above Cumming estimation plot. The raw data is plotted on the upper axes. On the lower axes, mean differences are plotted as bootstrap sampling distributions. Each mean difference is depicted as a dot. Each 95% confidence interval is indicated by the ends of the vertical error bars [see (30)]. Panels show **(A)** characteristic path length (measure of integration), **(B)** cluster coefficient (measure of segregation), **(C)** betweenness centrality (measure of centrality), and **(D)** closeness centrality (measure of centrality). *Denotes statistically significant differences at a significance level of 0.05 Bonferroni corrected.

Patients with schizophrenia did not show differences in measures of integration (characteristic path length and global efficiency) compared to healthy controls (Figure 1, Table 2). Patients with schizoaffective disorder showed an increase in characteristic path length, which, however, did not survive correction for multiple comparisons (*g* = 0.766, *p* = 0.266 Bonferroni corrected, statistical power = 0.36; see also Figure 1, Table 3) Note the small sample size of the schizoaffective group. We found no other significant correlations between measures of integration and PANSS total scores neither for the SCZ nor for the SCZaff group (see Supplementary Figures 1–8).

**Table 2:** Permutation test statistics for the SCZ group (sample size *n* = 43).

	**Hedge's g**	**95% CI interval**	**Permutation *p*-value**
			**(uncorrected)**
Characteristic path length	0.38	[−0.056, 0.814]	*p* = 0.079
Cluster coefficient	−0.406	[−0.715, −0.017]	*p* = 0.056
Betweenness centrality	−0.644	[−1.070, −0.204]	*p* = 0.004
Closeness centrality	−0.384	[−0.808, 0.059]	*p* = 0.078
Transitivity	−0.340	[−0.753, −0.091]	*p* = 0.110
Global efficiency	−0.381	[−0.802, 0.058]	*p* = 0.081
Assortativity	−0.198	[−0.605, 0.226]	*p* = 0.363

*Comparison against the HC group [sample size n = 43; see (30)]*.

**Table 3:** Permutation test statistics for the SCZaff group (sample size *n* = 9).

	**Hedge's g**	**95% CI interval**	**Permutation *p*-value**
			**(uncorrected)**
Characteristic path length	0.766	[−0.206, 1.480]	*p* = 0.038
Cluster coefficient	−0.371	[−0.770, 0.473]	*p* = 0.293
Betweenness centrality	−1.080	[−1.810, −0.049]	*p* = 0.005
Closeness centrality	−0.626	[−1.230, 0.429]	*p* = 0.090
Transitivity	−0.505	[−1.190, 0.678]	*p* = 0.173
Global efficiency	−0.615	[−1.230, 0.462]	*p* = 0.093
Assortativity	0.065	[−0.461, 0.642]	*p* = 0.855

*Comparison against the HC group [sample size n = 43; see (30)]*.

#### 3.1.2. Segregation

Patients from both groups did not show differences in measures of segregation (cluster coefficient and transitivity) compared to healthy controls (Figure 1, Table 2). we found a strong positive correlation between cluster coefficient and PANSS general score (*r* = 0.693, *p* = 0.038; see Supplementary Figure 7) and a moderate negative correlation between the cluster coefficient and the PANSS positive score for the SCZaff group (*r* = −0.304, *p* = 0.047; see Supplementary Figure 4). However, none of them survived correction for multiple comparisons.

#### 3.1.3. Centrality

**Figure 2:**
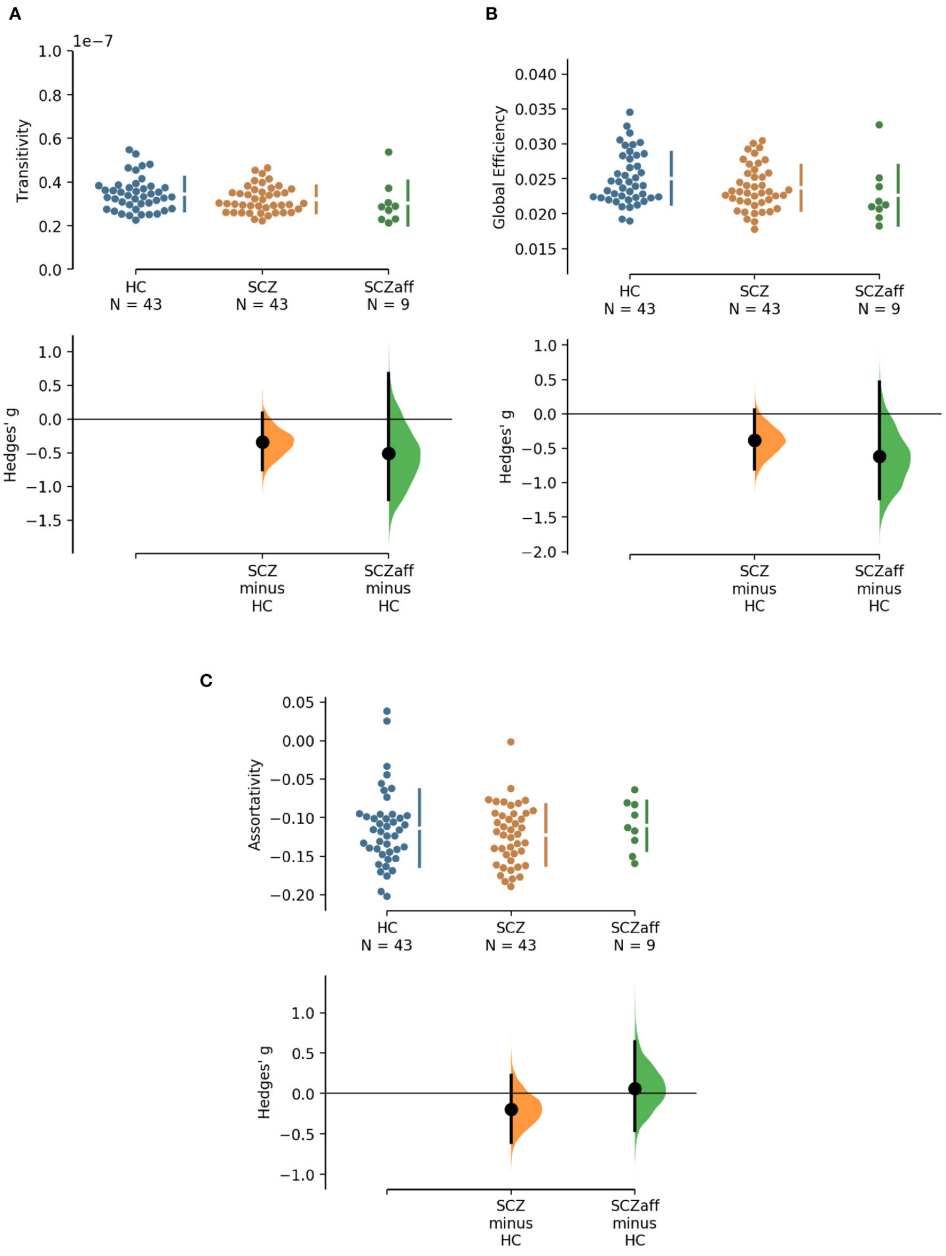
Hedge's *g* for the comparisons of various graph measures for the SCZ and SCZaff groups against the HC group are shown in the above Cumming estimation plot. The raw data is plotted on the upper axes. On the lower axes, mean differences are plotted as bootstrap sampling distributions. Each mean difference is depicted as a dot. Each 95% confidence interval is indicated by the ends of the vertical error bars [see (30)]. Panels show **(A)** transitivity (measure of segregation), **(B)** global efficiency (measure of integration), and **(C)** assortativity (measure of resilience).

Patients with schizophrenia showed a strong reduction of betweenness centrality compared to healthy controls (*g* = −0.644, *p* < 0.05 Bonferroni corrected, statistical power = 0.87; see also Figure 2, Table 2). In our exploratory analysis of the small schizoaffective group, patients showed a similar reduction in betweenness centrality than the SCZ group (*g* = −1.080, *p* < 0.05 Bonferroni corrected, statistical power = 0.65; see also Figure 2, Table 3). We found no significant correlations between centrality measures and PANSS total scores neither for the SCZ nor for the SCZaff group (see Supplementary Figures 1–8).

#### 3.1.4. Resilience

Patients from both groups did not show differences in network resilience measured by assortativity compared to healthy controls (Figure 2, Table 2). We found a moderate positive correlation between assortativity and PANSS total score in the SCZ group (*r* = 0.346, *p* = 0.023; see also Supplementary Figure 1) and a strong negative correlation between assortativity and PANSS positive score in the SCZaff group (*r* = −0.846, *p* = 0.004; see Supplementary Figure 2). However, none of them survived correction for multiple comparisons.

Since patients with schizophrenia and schizoaffective disorder are sometimes grouped together, we additionally performed the graph measure analysis for a single, pooled patient group with a “schizophrenia broad” categorization, which can be found in Supplementary Figures 14, 15, Supplementary Table 2. Unsurprisingly, we found a significant reduction of betweenness centrality in the pooled patient group compared to the healthy controls. We further explored a potential influence of the antipsychotic medication on the graph measures. We did not find any correlation between the medication dose (measured in CPZ-equivalent dose) and the graph measures used in this study for any of the patient groups (see Supplementary Tables 4, 5, respectively).

### 3.2. Controllability

Going beyond traditional network measures, we also explored two control theoretic measures: average and modal controllability. Network control theory has been increasingly utilized in network neuroscience and offers a mechanistic framework to understand the effects of local changes in dynamics on the global brain state (27). We specifically chose average and modal controllability because they offer information on two different aspects of controllability: while nodes with a high average controllability have the ability to convey large changes in network dynamics by moving the global system into many easily reachable states, nodes with high modal controllability can move the system to states that are difficult to reach (27).

**Figure 3:**
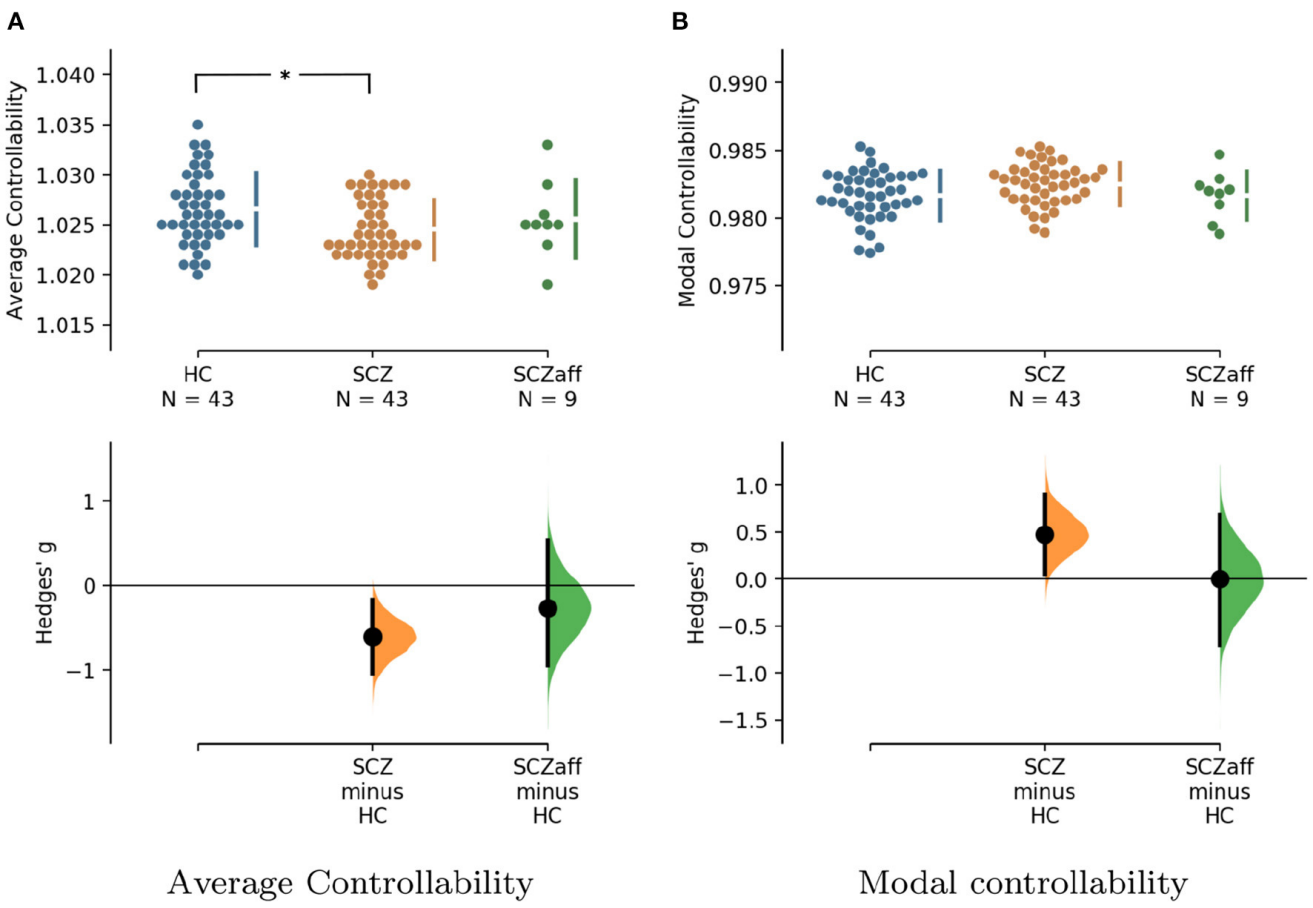
Hedge's *g* for the comparisons of **(A)** average and **(B)** modal controllability of weighted graphs of the SCZ and SCZaff groups against the HC group are shown in the above Cumming estimation plot. The raw data is plotted on the upper axes. On the lower axes, Hedge's *g* is plotted as bootstrap sampling distributions. Each *g* is depicted as a dot. Each 95% confidence interval is indicated by the ends of the vertical error bars [see (30)]. *Denotes statistically significant differences at a significance level of 0.05 Bonferroni corrected.

For the SCZ group we found a strong decrease in average controllability (*g* = −0.606, *p* < 0.05 Bonferroni corrected; see also Figure 3, Table 4). Additionally, we also found a moderate increase of modal controllability in the SCZ group, which, however, did not survive correction for multiple comparisons (*g* = 0.476, *p* = 0.056 Bonferroni corrected; see also Figure 3, Table 4). We found no differences for the SCZaff group compared to the healthy controls. Again, we pooled the two patient groups and performed the same analysis (see Supplementary Figure 16, Supplementary Table 3). We found a significant reduction of average controllability for this pooled patient group compared to the HC group.

**Table 4:** Permutation test statistics for the SCZaff group (sample size *n* = 9).

	**Hedge's g**	**95% CI**	**Permutation**
			***p*-value (uncorr.)**
Average controllability	−0.606	[−1.040, −0.171]	*p* = 0.006
(SCZ)			
Modal controllability	0.476	[0.047, 0.897]	*p* = 0.028
(SCZ)			
Average controllability	−0.265	[−0.944, 0.537]	*p* = 0.467
(SCZaff)			
Modal controllability	0.001	[−0.701, 0.686]	*p* = 0.994
(SCZaff)			

*Comparison against the HC group [sample size n = 43; see (30)]*.

We found no correlations between controllability measures and symptom scores, neither for the PANSS total score nor for the positive or negative subscores, in either of the patient groups (see Supplementary Figures 9–12). We again tested for a potential influence of the antipsychotic medication on the measures. We did not find any correlation between the medication dose (measured in CPZ-equivalent dose) and the control measures used here for any of the patient groups (see Supplementary Tables 4, 5, respectively).

**Figure 4:**
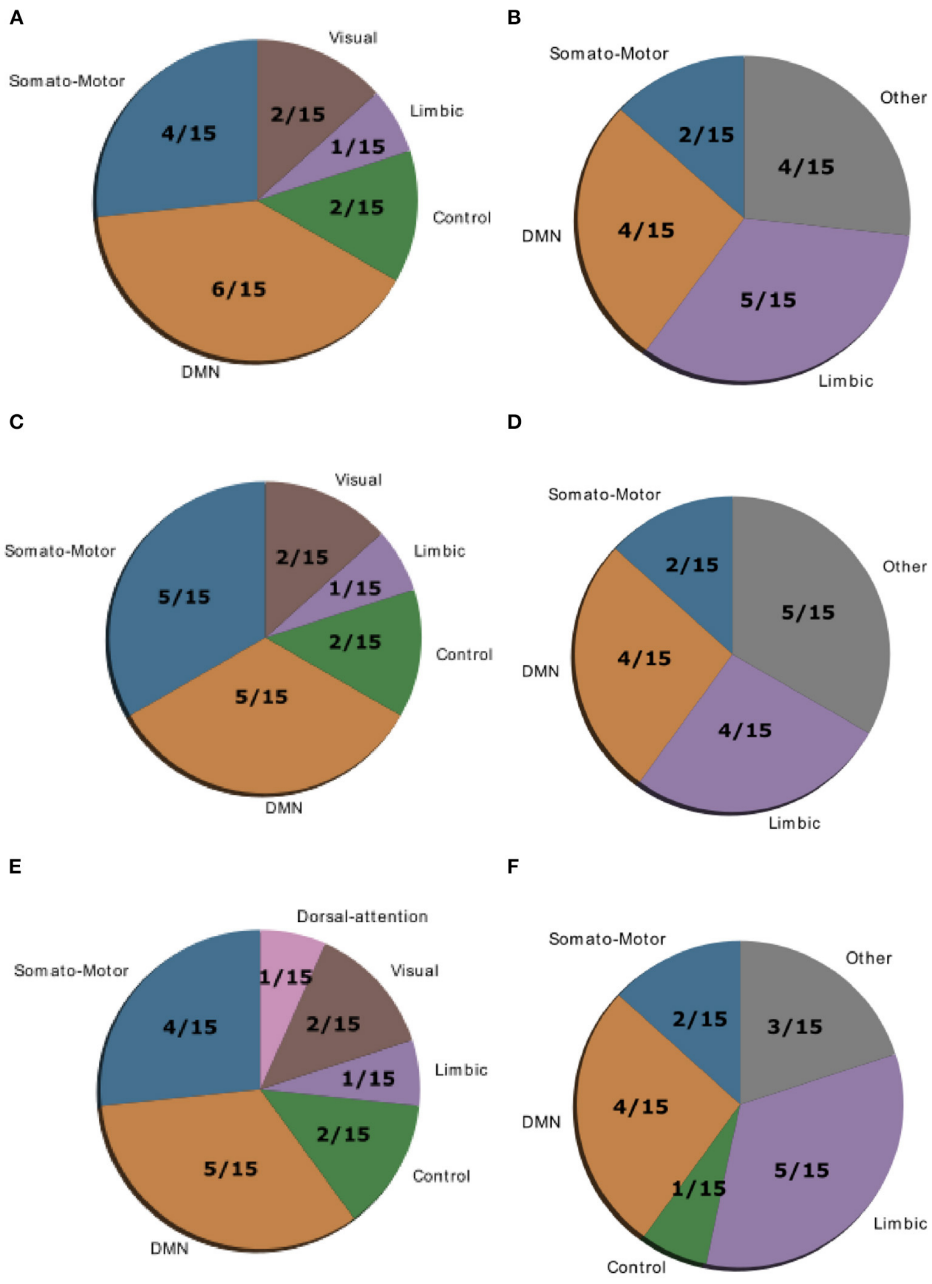
Location of cognitive control hubs for controllability across large-scale cognitive systems in the HC group [average controllability **(A)** and modal controllability **(B)**], the SCZ group [average controllability **(C)** and modal controllability **(D)**] and the SCZaff group [average controllability **(E)** and modal controllability **(F)**]. We chose the top 15 regions with highest average and modal controllability (averaged over all subjects), respectively.

Gu et al. (10) demonstrated that control regions are differentially distributed across cognitive systems depending on whether they show high average or high modal controllability. Therefore, we next asked whether this differential distribution would still be valid in both patient groups or not. To test this, we averaged controllability measures of all 94 regions of the AAL2 atlas (for both average and modal controllability) across all subjects of a group and then extracted the 15 regions with the highest measures. These regions were then assigned one of the following 8 large-scale cognitive systems based on the 7 functional cortical networks from Yeo et al. (31) but extended to also include subcortical structures: Somatomotor, Default Mode (DMN), Control, Visual, Dorsal Attention, Ventral Attention, Limbic, and Other [subcortical regions that could not be assigned to any system; see Supplementary Tables 2–4 for the assignments and (10) for more details on the approach]. We found that for average controllability most regions belong to the DMN followed by the somatomotor network in all three groups (Figure 4), consistent with (10). For modal controllability we found that in all three groups most regions belong to the limbic category (Figure 4). Interestingly, again for all three groups, DMN network regions also made up a large share of the top modal controllability regions. Importantly, the DMN regions with high modal controllability here where distinct from the DMN regions with high average controllability, highlighting the importance, and uniqueness of the DMN. Nevertheless, the main finding here is that there are no substantial changes in regions with high average/modal controllability between control and patient groups. There were no strong differences between the mean controllability of the top 15 regions of the different groups, neither for average nor for modal controllability (Supplementary Figure 13, Supplementary Table 1).

## 4. Discussion

From a dynamical systems perspective, the brain can be viewed as an interconnected network in which complex behaviors are characterized by transitions between network states, i.e., spatio-temporal patterns of activity. Psychiatric disorders have been associated with disturbances in these dynamical interactions between brain regions. Schizophrenia for example has been characterized as a disorder of dysconnection (2), where an overall reduction in connectivity leads to disruptions of normal cognitive functions. In this study, we apply techniques from network theory, i.e., standard graph measures, together with novel methods from control theory, i.e., controllability measures, to characterize the degree of dysconnection in structural brain networks derived from white matter fibre connectivity in patients with schizophrenia and a small sample of patients with schizoaffective disorder.

### Integration, Segregation, Centrality, and Resilience

We found a reduction in betweenness centrality in both patients with schizophrenia and schizoaffective disorder. Since nodes with high betweenness centrality, which quantifies the number of times a node acts as a bridge along shortest paths between pairs of nodes, reflect nodes which can potentially exert strong control over the activity and information flow in the network, the reduction in the patient groups suggests a less controlled flow and a general decentralization of the brain network. We did not find any significant differences between the patient groups and the healthy control group for integration, segregation or resilience measures.

Several studies have analyzed differences in the structural connectome of patients with schizophrenia using network measures. Overall, these studies report an altered connectome with altered integration and centrality. Filippi et al. (9) found that patient networks were characterized by longer communication pathways and fewer central hubs compared to healthy controls. A reduction in hub structure, especially the hub hierarchy, was also found in a study by Bassett et al. (6). Furthermore, altered path length between brain nodes and reduced efficiency have consistently been found in schizophrenia (6, 23, 32, 33), with frontal and parietal lobes being most effected. Frontal and parietal lobes were also the areas with the highest reduction of connectivity strength (34). Overall, our findings are consistent with these previous studies. The reduced betweenness centrality we see would imply a reduction of centrality hubs and their hierarchy, as found by Filippi et al. (9) and Bassett et al. (6) and distort normal communication pathways.

### Network Control

Structural brain networks from patients with schizophrenia display a strongly reduced average controllability. This finding implies that the dysconnectivity in patients with schizophrenia results in a reduced capacity of individual nodes to steer the network activity from one brain state to another. This finding is also consistent with the observation that random state switching is more frequent in schizophrenic models, since the lower average controllability implies a reduced capacity of keeping the system in a desired state, which makes random transitions more likely. The strong decrease of average controllability in schizophrenia patients would be expected in a network where the central hub structure is disturbed, as discussed above.

Studies show that the affective symptom components in bipolar disorder and schizoaffective disorder are relatively similar. Therefore, it is interesting that Collin et al. (17) found the central hub structure to be intact in patients with bipolar disorder. A finding which might explain the absence of differences in average controllability between our schizoaffective patient group and healthy controls, because of the link between central structural hubs, i.e., influential high-degree nodes, and their ability to easily push the brain network into different brain states reflected in their average controllability. However, this absence might also be a consequence of the patient sample consisting of stably medicated subjects, since Wang et al. (35) report a disruption of the rich-club hub system in bipolar disorder. Antipsychotic medication has been shown to alter connectivity in SCZ depending on the type of antipsychotic (36). Furthermore, mood stabilizers tended to renormalize connectivity, while antipsychotic medication did not in patients with bipolar disorder (37). Therefore, differences in medication in our patient samples might explain the absence of differences with respect to average controllability and central hub structure. However, we have to note that the small sample size and the resulting lack of statistical power could also explain this absence.

Last, the distribution of large-scale functional cognitive systems across nodes with high average or modal controllability is not altered in patient groups, suggesting a global reduction of controllability that does not affect a specific cognitive system. This is not surprising, given that patients with schizophrenia suffer from an array of cognitive symptoms.

### Limitations

Notably, the current study has its limitations. First, by restricting ourselves to the anatomical parcellation given by the AAL2 atlas, we commit to a specific mapping between MR voxels and brain regions. Importantly, in terms of parcellation schemes, there are a variety of options and no standard has been established so far. Taking into account the fact that these schemes differ not only with regard to the number of regions in which they divide the brain, but also with regard to other key aspects, such as the exact location of the inter-regional borders or the method through which they were generated, it is possible that the specific choice of the parcellation scheme has an impact on subsequent graph theoretical findings. Therefore, a reproduction of our findings with other parcellation schemes seems warranted. Second, the patient sample considered in this study consists of chronic, stably medicated subjects and therefore, the findings can only be considered representative of this particular subpopulation of patients. It would indeed be very interesting to track changes in network and controllability measures in other patient subpopulations such as first-episode psychosis subjects or throughout the course of the disorders in a longitudinal study design. The presented analysis cannot address whether our findings reflect a primary change due to the disorder or whether they are indeed caused by the antipsychotic medication, even though the medication dosage did not correlate with any of the graph or control measures analyzed here. Last, the sample size of the current study is not very large and therefore, the results should be confirmed in a replication study with a larger patient group. Importantly, the results from the patients with schizoaffective disorder should be treated with caution because of the very small number of subjects.

This study is, to our best knowledge, the first study investigating controllability measures in patients with schizophrenia and schizoaffective disorder and it corroborates previous findings of altered structural connectivity and suggests that a control theoretic approach could be useful in pathological research.

## Data Availability Statement

Publicly available datasets were analyzed in this study. This data can be found at: schizconnect.org.

## Ethics Statement

The studies involving human participants were reviewed and approved by Institutional Review Board University of New Mexico. The patients/participants provided their written informed consent to participate in this study.

## Author Contributions

CD and CM designed the study and wrote the manuscript. CD, SG, FK, and SF processed the data. CD, SG, and CM analyzed the data. CD, CM, and KO discussed the findings. All authors provided critical feedback to the manuscript.

## Conflict of Interest

The authors declare that the research was conducted in the absence of any commercial or financial relationships that could be construed as a potential conflict of interest.

## References

[1] Bullmore E Frangou S Murray R. The dysplastic net hypothesis: an integration of developmental and dysconnectivity theories of schizophrenia. Schizophr Res. (1997) 28:143–56. 10.1016/S0920-9964(97)00114-X 9468349

[2] Friston KJ. The disconnection hypothesis. Schizophr Res. (1998) 30:115–25. 10.1016/S0920-9964(97)00140-0 9549774

[3] Brandl F Avram M Weise B Shang J Simoes B Bertram T. Specific substantial dysconnectivity in schizophrenia: a transdiagnostic multimodal meta-analysis of resting-state functional and structural magnetic resonance imaging studies. Biol Psychiatry. (2019) 85:573–83. 10.1016/j.biopsych.2018.12.003 30691673

[4] Li S Hu N Zhang W Tao B Dai J Gong Y. Dysconnectivity of multiple brain networks in schizophrenia: a meta-analysis of resting-state functional connectivity. Front Psychiatry. (2019) 10:482. 10.3389/fpsyt.2019.00482 31354545 PMC6639431

[5] Sporns O Chialvo DR Kaiser M Hilgetag CC. Organization, development and function of complex brain networks. Trends Cogn Sci. (2004) 8:418–25. 10.1016/j.tics.2004.07.008 15350243

[6] Bassett DS Bullmore E Verchinski BA Mattay VS Weinberger DR Meyer-Lindenberg A. Hierarchical organization of human cortical networks in health and schizophrenia. J Neurosci. (2008) 28:9239–48. 10.1523/JNEUROSCI.1929-08.2008 18784304 PMC2878961

[7] Bassett DS Bullmore ET Meyer-Lindenberg A Apud JA Weinberger DR Coppola R. Cognitive fitness of cost-efficient brain functional networks. Proc Natl Acad Sci USA. (2009) 106:11747–52. 10.1073/pnas.0903641106 19564605 PMC2703669

[8] Liu Y Liang M Zhou Y He Y Hao Y Song M. Disrupted small-world networks in schizophrenia. Brain. (2008) 131:945–61. 10.1093/brain/awn018 18299296

[9] Filippi M van den Heuvel MP Fornito A He Y Pol HEH Agosta F. Assessment of system dysfunction in the brain through MRI-based connectomics. Lancet Neurol. (2013) 12:1189–99. 10.1016/S1474-4422(13)70144-3 24120645

[10] Gu S Pasqualetti F Cieslak M Telesford QK Alfred BY Kahn AE. Controllability of structural brain networks. Nat Commun. (2015) 6:1–10. 10.1038/ncomms9414 PMC4600713 26423222

[11] Deco G Jirsa VK. Ongoing cortical activity at rest: criticality, multistability, and ghost attractors. J Neurosci. (2012) 32:3366–75. 10.1523/JNEUROSCI.2523-11.2012 22399758 PMC6621046

[12] Sporns O. Structure and function of complex brain networks. Dialog Clin Neurosci. (2013) 15:247. 10.31887/DCNS.2013.15.3/osporns 24174898 PMC3811098

[13] Cabral J Kringelbach ML Deco G. Functional connectivity dynamically evolves on multiple time-scales over a static structural connectome: models and mechanisms. NeuroImage. (2017) 160:84–96. 10.1016/j.neuroimage.2017.03.045 28343985

[14] Cocchi L Zalesky A Fornito A Mattingley JB. Dynamic cooperation and competition between brain systems during cognitive control. Trends Cogn Sci. (2013) 17:493–501. 10.1016/j.tics.2013.08.006 24021711

[15] Deco G Jirsa VK McIntosh AR. Emerging concepts for the dynamical organization of resting-state activity in the brain. Nat Rev Neurosci. (2011) 12:43–56. 10.1038/nrn2961 21170073

[16] Bora E Pantelis C. Meta-analysis of cognitive impairment in first-episode bipolar disorder: comparison with first-episode schizophrenia and healthy controls. Schizophr Bull. (2015) 41:1095–104. 10.1093/schbul/sbu198 25616505 PMC4535631

[17] Collin G van den Heuvel MP Abramovic L Vreeker A de Reus MA van Haren NE. Brain network analysis reveals affected connectome structure in bipolar I disorder. Hum Brain Mapp. (2016) 37:122–34. 10.1002/hbm.23017 26454006 PMC5597048

[18] Kay SR Opler LA Lindenmayer JP. The positive and negative syndrome scale (PANSS): rationale and standardisation. Br J Psychiatry. (1989) 155:59–65. 10.1192/S0007125000291514 2619982

[19] Aine C Bockholt HJ Bustillo JR Canive JM Caprihan A Gasparovic C. Multimodal neuroimaging in schizophrenia: description and dissemination. Neuroinformatics. (2017) 15:343–64. 10.1007/s12021-017-9338-9 28812221 PMC5671541

[20] Cetin MS Christensen F Abbott CC Stephen JM Mayer AR Canive JM. Thalamus and posterior temporal lobe show greater inter-network connectivity at rest and across sensory paradigms in schizophrenia. Neuroimage. (2014) 97:117–26. 10.1016/j.neuroimage.2014.04.009 24736181 PMC4087193

[21] Rolls ET Joliot M Tzourio-Mazoyer N. Implementation of a new parcellation of the orbitofrontal cortex in the automated anatomical labeling atlas. Neuroimage. (2015) 122:1–5. 10.1016/j.neuroimage.2015.07.075 26241684

[22] Behrens TE Berg HJ Jbabdi S Rushworth MF Woolrich MW. Probabilistic diffusion tractography with multiple fibre orientations: what can we gain? Neuroimage. (2007) 34:144–55. 10.1016/j.neuroimage.2006.09.018 17070705 PMC7116582

[23] Wang Q Su TP Zhou Y Chou KH Chen IY Jiang T. Anatomical insights into disrupted small-world networks in schizophrenia. Neuroimage. (2012) 59:1085–93. 10.1016/j.neuroimage.2011.09.035 21963918

[24] Achard S Bullmore E. Efficiency and cost of economical brain functional networks. PLoS Comput Biol. (2007) 3:e17. 10.1371/journal.pcbi.0030017 17274684 PMC1794324

[25] Gong G Rosa-Neto P Carbonell F Chen ZJ He Y Evans AC. Age-and gender-related differences in the cortical anatomical network. J Neurosci. (2009) 29:15684–93. 10.1523/JNEUROSCI.2308-09.2009 20016083 PMC2831804

[26] Rubinov M Sporns O. Complex network measures of brain connectivity: uses and interpretations. Neuroimage. (2010) 52:1059–69. 10.1016/j.neuroimage.2009.10.003 19819337

[27] Muldoon SF Pasqualetti F Gu S Cieslak M Grafton ST Vettel JM. Stimulation-based control of dynamic brain networks. PLoS Comput Biol. (2016) 12:e1005076. 10.1371/journal.pcbi.1005076 27611328 PMC5017638

[28] Marx B Koenig D Georges D. Optimal sensor and actuator location for descriptor systems using generalized gramians and balanced realizations. In: Proceedings of the 2004 American Control Conference. Vol. 3. (2004). p. 2729–34. 10.23919/ACC.2004.1383878

[29] Hamdan A Nayfeh A. Measures of modal controllability and observability for first-and second-order linear systems. J Guid Control Dyn, Reston, VA. (1989) 12:421–8. 10.2514/3.20424

[30] Ho J Tumkaya T Aryal S Choi H Claridge-Chang A. Moving beyond P values: everyday data analysis with estimation plots. Nat Methods. (2019) 16:565–6. 10.1038/s41592-019-0470-3 31217592

[31] Yeo BT Krienen FM Sepulcre J Sabuncu MR Lashkari D Hollinshead M. The organization of the human cerebral cortex estimated by intrinsic functional connectivity. J Neurophysiol. (2011) 106:1125–65. 10.1152/jn.00338.2011 21653723 PMC3174820

[32] Van Den Heuvel MP Pol HEH. Exploring the brain network: a review on resting-state fMRI functional connectivity. Eur Neuropsychopharmacol. (2010) 20:519–34. 10.1016/j.euroneuro.2010.03.008 20471808

[33] van den Heuvel MP Fornito A. Brain networks in schizophrenia. Neuropsychol Rev. (2014) 24:32–48. 10.1007/s11065-014-9248-7 24500505

[34] Zalesky A Fornito A Seal ML Cocchi L Westin CF Bullmore ET. Disrupted axonal fiber connectivity in schizophrenia. Biol Psychiatry. (2011) 69:80–9. 10.1016/j.biopsych.2010.08.022 21035793 PMC4881385

[35] Wang Y Deng F Jia Y Wang J Zhong S Huang H. Disrupted rich club organization and structural brain connectome in unmedicated bipolar disorder. Psychol Med. (2019) 49:510. 10.1017/S0033291718001150 29734951

[36] Leroux E Vandevelde A Tréhout M Dollfus S. Abnormalities of fronto-subcortical pathways in schizophrenia and the differential impacts of antipsychotic treatment: a DTI-based tractography study. Psychiatry Res. (2018) 280:22–9. 10.1016/j.pscychresns.2018.08.008 30145382

[37] Hafeman DM Chang KD Garrett AS Sanders EM Phillips ML. Effects of medication on neuroimaging findings in bipolar disorder: an updated review. Bipolar Disord. (2012) 14:375–410. 10.1111/j.1399-5618.2012.01023.x 22631621

