# Supplementary material for: Structural Differences Between Healthy Subjects and Patients With Schizophrenia or Schizoaffective Disorder: A Graph and Control Theoretical Perspective
Source: Front Psychiatry. 2021 Jun 28;12:669783. doi: 10.3389/fpsyt.2021.669783 (PMC8273511; doi:10.3389/fpsyt.2021.669783)
Supplement: Supplementary file 1 [file Data_Sheet_1.pdf]

Supplementary Material

1 SUPPLEMENTARY TABLES AND FIGURES

1.1 Tables

Table S1. Permutation test statistics for a comparison of the top 15 regions with highest average and modal controllability per group (SCZ and SCZaff), respectively, against the HC group.

	Hedge's g	95% CI interval	permutation p-value
Average controllability (SCZ)	-0.302	$[-1.030, 0.455]$	$p = 0.4$
Modal controllability (SCZ)	0.111	$[-0.580, 0.858]$	$p = 0.741$
Average controllability (SCZaff)	-0.054	$[-0.824, 0.677]$	$p = 0.877$
Modal controllability (SCZaff)	0.21	$[-0.487, 0.922]$	$p = 0.555$

Table S2. Permutation test statistics for the pooled SCZ and SCZaff group (sample size $n = 52$). Comparison against the HC group (sample size $n = 43$).

	Hedge's g	95% CI interval	permutation p-value (uncorrected)
Characteristic path length	0.447	$[0.030, 0.866]$	$p = 0.034$
Cluster coefficient	-0.422	$[-0.739, -0.038]$	$p = 0.032$
Betweenness centrality	-0.712	$[-1.130, -0.294]$	$p = 0.001$
Closeness centrality	-0.429	$[-0.852, -0.015]$	$p = 0.040$
Transitivity	-0.368	$[-0.787, 0.034]$	$p = 0.082$
Global efficiency	-0.424	$[-0.857, -0.009]$	$p = 0.043$
Assortativity	-0.155	$[-0.550, 0.262]$	$p = 0.448$

Table S3. Permutation test statistics for the pooled SCZ and SCZaff group (sample size $n = 52$). Comparison against the HC group (sample size $n = 43$).

	Hedge's g	95% CI	permutation p-value (uncorr.)
Average controllability	-0.606	$[-1.040, -0.171]$	$p = 0.006$
Modal controllability	0.476	$[0.047, 0.897]$	$p = 0.028$

Table S4. Correlation of medication (CPZ equivalent dose) with the graph and controllability measures for the SCZ group (sample size $n = 43$).

	Correlation coefficient	p-value
Characteristic path length	0.124	$p = 0.428$
Cluster coefficient	-0.106	$p = 0.499$
Betweenness centrality	0.021	$p = 0.893$
Closeness centrality	-0.012	$p = 0.939$
Transitivity	0.077	$p = 0.624$
Global efficiency	-0.018	$p = 0.909$
Assortativity	-0.017	$p = 0.914$
Average Controllability	-0.191	$p = 0.220$
Modal Controllability	0.183	$p = 0.240$

Table S5. Correlation of medication (CPZ equivalent dose) with the graph and controllability measures for the SCZaff group (sample size $n = 9$).

	Correlation coefficient	p-value
Characteristic path length	-0.539	$p = 0.134$
Cluster coefficient	0.044	$p = 0.911$
Betweenness centrality	0.263	$p = 0.494$
Closeness centrality	0.461	$p = 0.212$
Transitivity	-0.375	$p = 0.320$
Global efficiency	0.462	$p = 0.211$
Assortativity	-0.002	$p = 0.996$
Average Controllability	0.309	$p = 0.418$
Modal Controllability	-0.239	$p = 0.536$

Table S6. Assignment of AAL2 regions to one of 8 cognitive systems: Somatomotor, Default Mode (DMN), Control, Visual, Dorsal Attention, Ventral Attention, Limbic, and Other.

Region	Cognitive System
Precentral_L	Somatomotor
Precentral_R	Somatomotor
Frontal_Sup_2_L	Default Mode Network
Frontal_Sup_2_R	Default Mode Network
Frontal_Mid_2_L	Control
Frontal_Mid_2_R	Control
Frontal_Inf_Oper_L	Ventral Attention
Frontal_Inf_Oper_R	Control
Frontal_Inf_Tri_L	Control
Frontal_Inf_Tri_R	Control
Frontal_Inf_Orb_2_L	Default Mode Network
Frontal_Inf_Orb_2_R	Default Mode Network
Rolandic_Oper_L	Somatomotor
Rolandic_Oper_R	Somatomotor
Supp_Motor_Area_L	Somatomotor
Supp_Motor_Area_R	Somatomotor
Olfactory_L	Limbic
Olfactory_R	Limbic
Frontal_Sup_Medial_L	Default Mode Network
Frontal_Sup_Medial_R	Default Mode Network
Frontal_Med_Orb_L	Default Mode Network
Frontal_Med_Orb_R	Default Mode Network
Rectus_L	Limbic
Rectus_R	Limbic
OFCmed_L	Limbic
OFCmed_R	Limbic
OFCant_L	Limbic
OFCant_R	Control
OFCpost_L	Limbic
OFCpost_R	Limbic
OFClat_L	Default Mode Network
OFClat_R	Default Mode Network
Insula_L	Ventral Attention
Insula_R	Ventral Attention
Cingulate_Ant_L	Default Mode Network
Cingulate_Ant_R	Default Mode Network
Cingulate_Mid_L	Ventral Attention
Cingulate_Mid_R	Ventral Attention
Cingulate_Post_L	Default Mode Network
Cingulate_Post_R	Default Mode Network
Hippocampus_L	Other
Hippocampus_R	Limbic
ParaHippocampal_L	Limbic
ParaHippocampal_R	Limbic
Amygdala_L	Limbic
Amygdala_R	Other
Calcarine_L	Visual
Calcarine_R	Visual
Cuneus_L	Visual
Cuneus_R	Visual
Lingual_L	Visual
Lingual_R	Visual

Table S7. Table S6, continued. Assignment of AAL2 regions to one of cognitive systems continued: Somatomotor, Default Mode (DMN), Control, Visual, Dorsal Attention, Ventral Attention, Limbic, and Other.

Region	Cognitive System
Occipital_Sup_L	Visual
Occipital_Sup_R	Visual
Occipital_Mid_L	Visual
Occipital_Mid_R	Visual
Occipital_Inf_L	Visual
Occipital_Inf_R	Visual
Fusiform_L	Visual
Fusiform_R	Visual
Postcentral_L	Somatomotor
Postcentral_R	Somatomotor
Parietal_Sup_L	Dorsal Attention
Parietal_Sup_R	Dorsal Attention
Parietal_Inf_L	Dorsal Attention
Parietal_Inf_R	Control
SupraMarginal_L	Ventral Attention
SupraMarginal_R	Ventral Attention
Angular_L	Default Mode Network
Angular_R	Default Mode Network
Precuneus_L	Default Mode Network
Precuneus_R	Dorsal Attention
Paracentral_Lobule_L	Somatomotor
Paracentral_Lobule_R	Somatomotor
Caudate_L	Limbic
Caudate_R	Other
Putamen_L	Other
Putamen_R	Ventral Attention
Pallidum_L	Other
Pallidum_R	Other
Thalamus_L	Other
Thalamus_R	Other
Heschl_L	Somatomotor
Heschl_R	Somatomotor
Temporal_Sup_L	Somatomotor
Temporal_Sup_R	Somatomotor
Temporal_Pole_Sup_L	Limbic
Temporal_Pole_Sup_R	Limbic
Temporal_Mid_L	Default Mode Network
Temporal_Mid_R	Default Mode Network
Temporal_Pole_Mid_L	Limbic
Temporal_Pole_Mid_R	Limbic
Temporal_Inf_L	Limbic
Temporal_Inf_R	Limbic

1.2 Figures

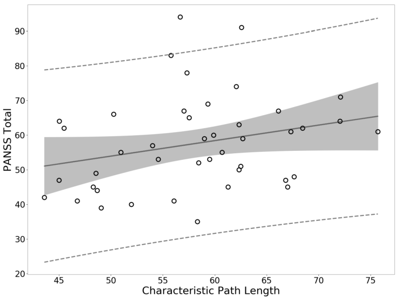

(a) $r = 0.266$, $p = 0.085$

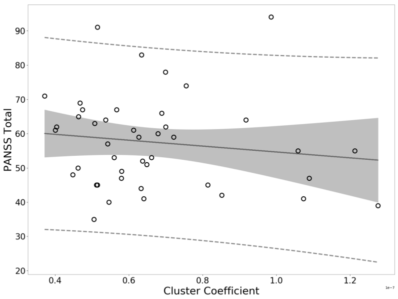

(b) $r = -0.140$, $p = 0.372$

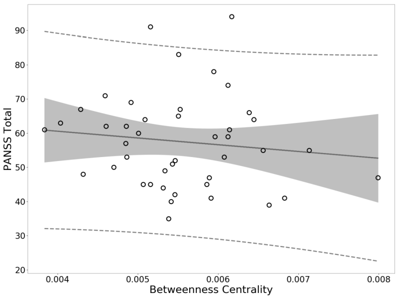

(c) $r = -0.123$, $p = 0.432$

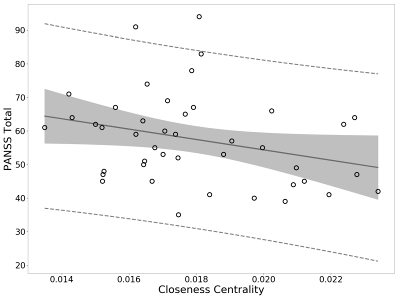

(d) $r = -0.290$, $p = 0.059$

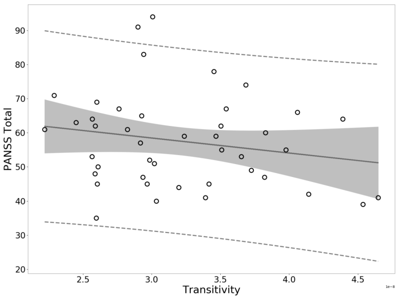

(e) $r = -0.198$, $p = 0.202$

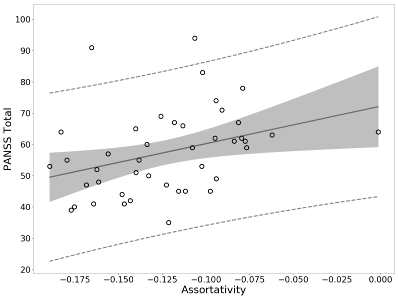

(f) $r = 0.346$, $p = 0.023$

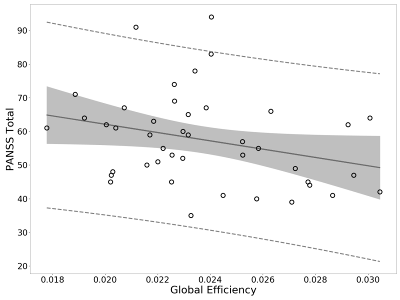

(g) $r = -0.290$, $p = 0.059$

Figure S1: Correlation of graph measures of the SCZ group with PANSS total scores. a) characteristic path length, b) cluster coefficient, c) betweenness centrality, d) closeness centrality, e) transitivity, f) assortativity, and g) global efficiency. The line corresponds to the fitted trend line and the shaded region the 95% confidence interval.

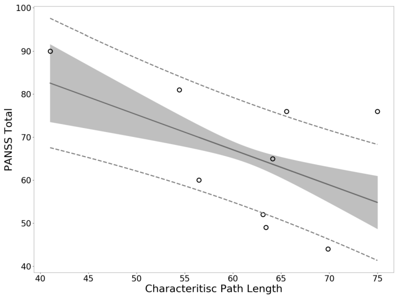

(a) $r = -0.508$, $p = 0.163$

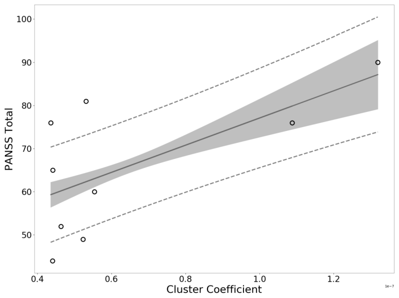

(b) $r = 0.647$, $p = 0.060$

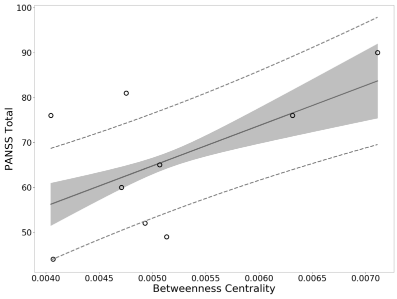

(c) $r = 0.565$, $p = 0.113$

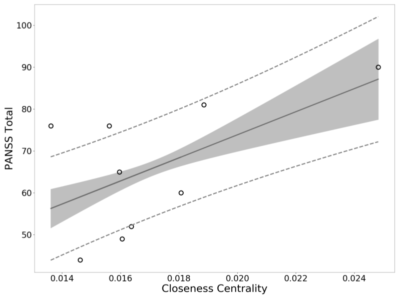

(d) $r = 0.573$, $p = 0.107$

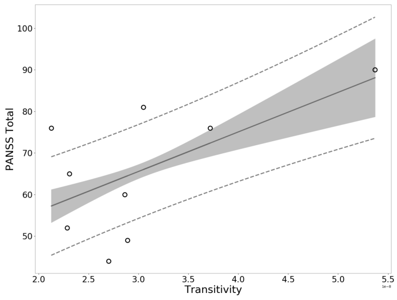

(e) $r = 0.600$, $p = 0.087$

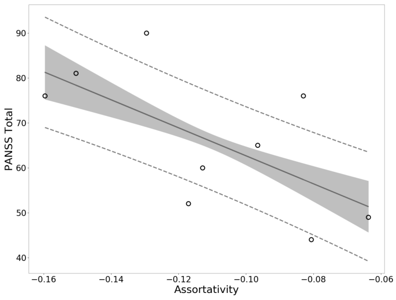

(f) $r = -0.639$, $p = 0.064$

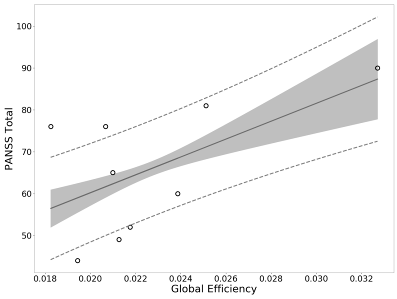

(g) $r = 0.580$, $p = 0.102$

Figure S2: Correlation of graph measures of the SCZaff group with PANSS total scores. a) characteristic path length, b) cluster coefficient, c) betweenness centrality, d) closeness centrality, e) transitivity, f) assortativity, and g) global efficiency. The line corresponds to the fitted trend line and the shaded region the 95% confidence interval.

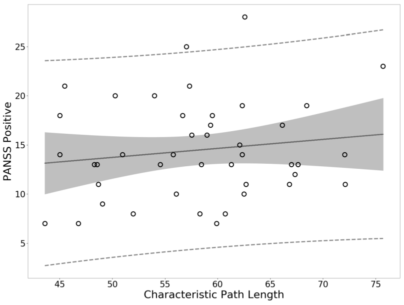

(a) $r = 0.148, p = 0.342$

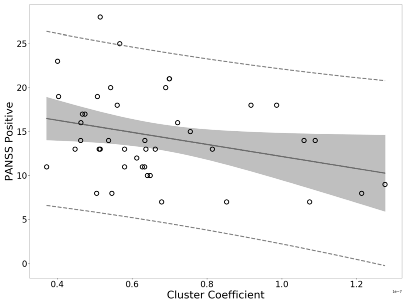

(b) $r = -0.304$, $p = 0.047$

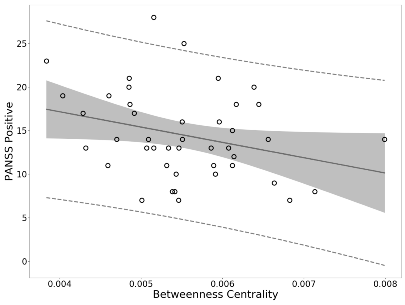

(c) $r = -0.299$, $p = 0.051$

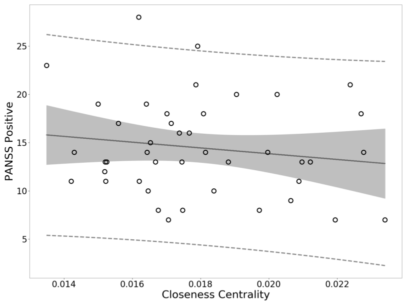

(d) $r = -0.153$, $p = 0.327$

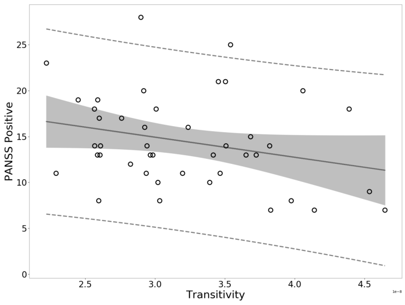

(e) $r = -0.269$, $p = 0.081$

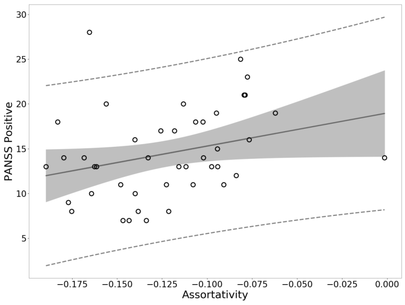

(f) $r = 0.291$, $p = 0.058$

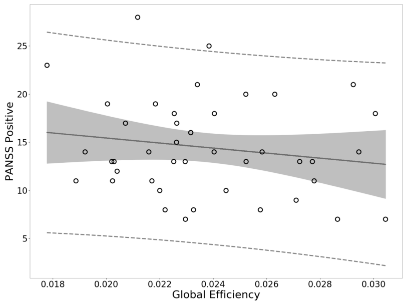

(g) $r = -0.168$, $p = 0.282$

Figure S3: Correlation of graph measures of the SCZ group with PANSS positive scores.a) characteristic path length, b) cluster coefficient, c) betweenness centrality, d) closeness centrality, e) transitivity, f) assortativity, and g) global efficiency. The line corresponds to the fitted trend line and the shaded region the 95% confidence interval.

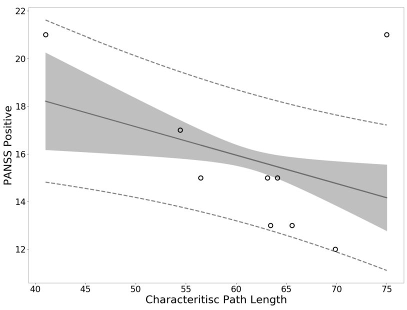

(a) $r = -0.355$, $p = 0.348$

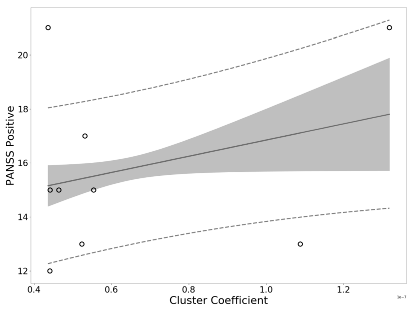

(b) $r = 0.294$, $p = 0.441$

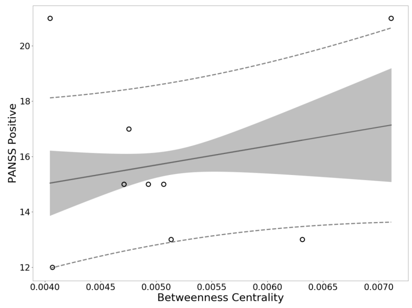

(c) $r = 0.207$, $p = 0.593$

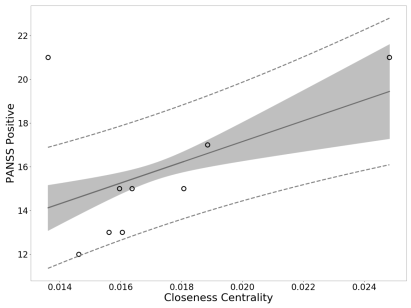

(d) $r = 0.474$, $p = 0.198$

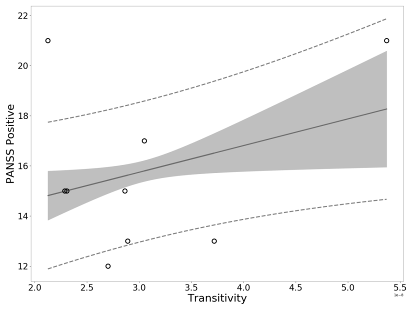

(e) $r = 0.322$, $p = 0.398$

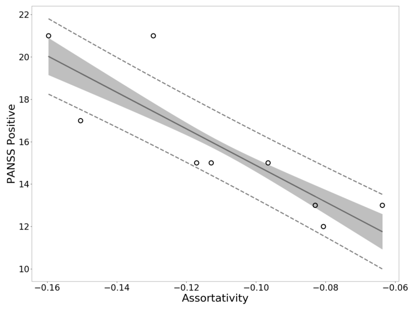

(f) $r = -0.846$, $p = 0.004$

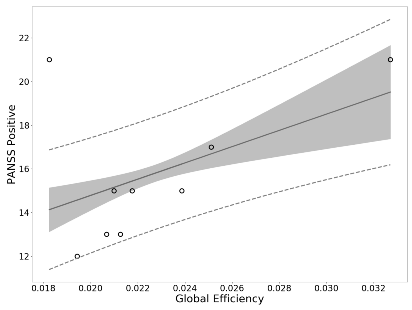

(g) $r = 0.484$, $p = 0.187$

Figure S4: Correlation of graph measures of the SCZaff group with PANSS positive scores. a) characteristic path length, b) cluster coefficient, c) betweenness centrality, d) closeness centrality, e) transitivity, f) assortativity, and g) global efficiency. The line corresponds to the fitted trend line and the shaded region the 95% confidence interval.

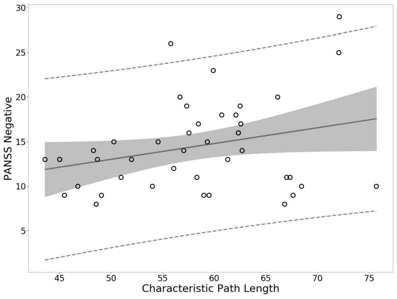

(a) $r = 0.285$, $p = 0.064$

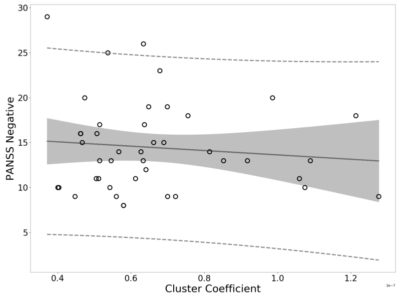

(b) $r = -0.107$, $p = 0.494$

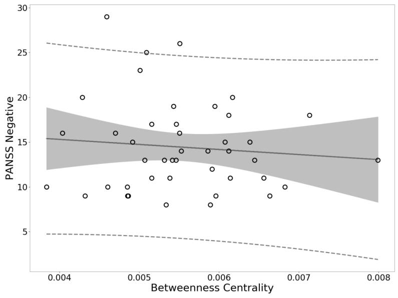

(c) $r = -0.095$, $p = 0.544$

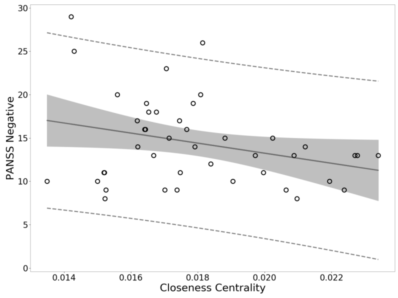

(d) $r = -0.296$, $p = 0.054$

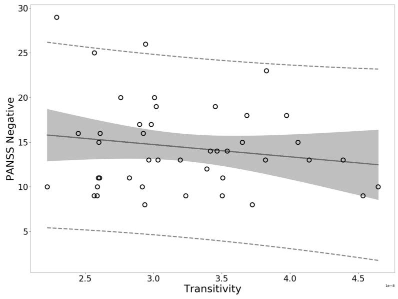

(e) $r = -0.168$, $p = 0.283$

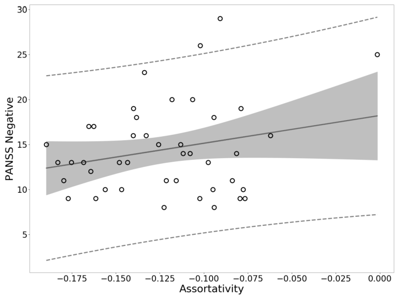

(f) $r = 0.242$, $p = 0.118$

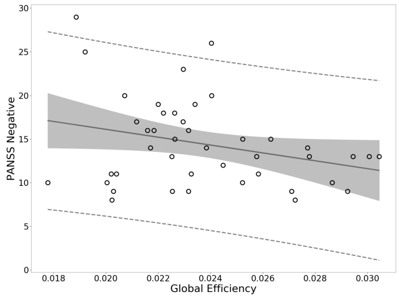

(g) $r = -0.288$, $p = 0.061$

Figure S5: Correlation of graph measures of the SCZ group with PANSS negative scores. a) characteristic path length, b) cluster coefficient, c) betweenness centrality, d) closeness centrality, e) transitivity, f) assortativity, and g) global efficiency. The line corresponds to the fitted trend line and the shaded region the 95% confidence interval.

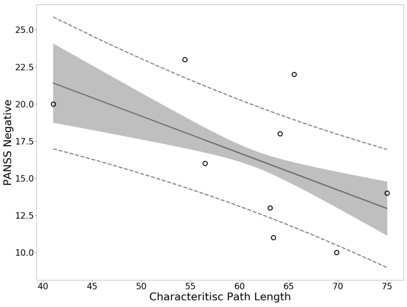

(a) $r = -0.520$, $p = 0.151$

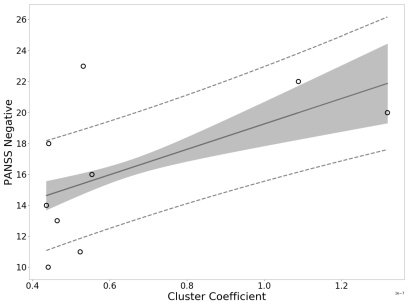

(b) $r = 0.566$, $p = 0.112$

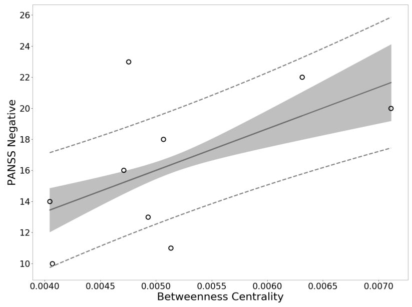

(c) $r = 0.568$, $p = 0.111$

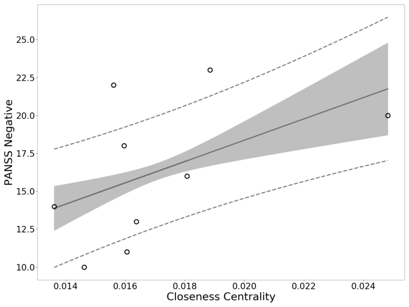

(d) $r = 0.491$, $p = 0.179$

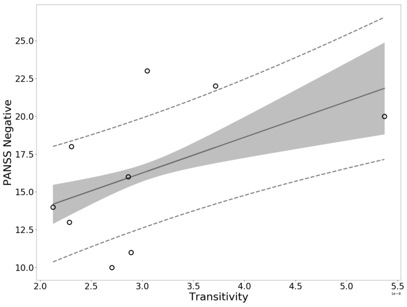

(e) $r = 0.500$, $p = 0.170$

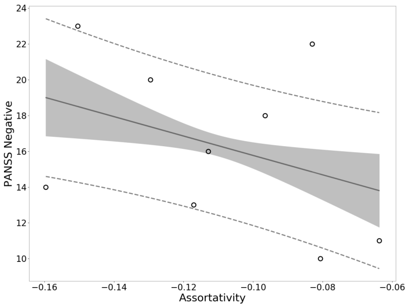

(f) $r = -0.374$, $p = 0.322$

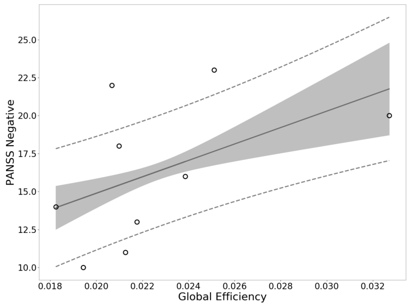

(g) $r = 0.493$, $p = 0.178$

Figure S6: Correlation of graph measures of the SCZaff group with PANSS negative scores. a) characteristic path length, b) cluster coefficient, c) betweenness centrality, d) closeness centrality, e) transitivity, f) assortativity, and g) global efficiency. The line corresponds to the fitted trend line and the shaded region the 95% confidence interval.

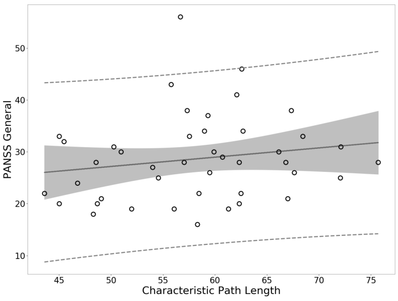

(a) $r = 0.174$, $p = 0.265$

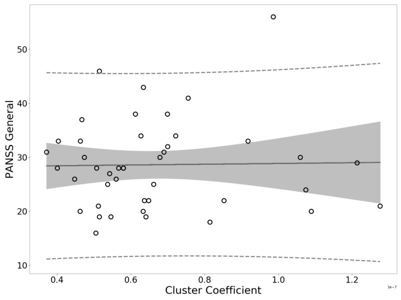

(b) $r = -0.019$, $p = 0.905$

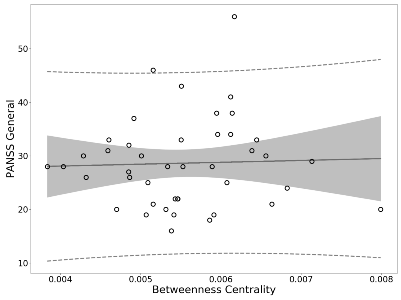

(c) $r = -0.036$, $p = 0.821$

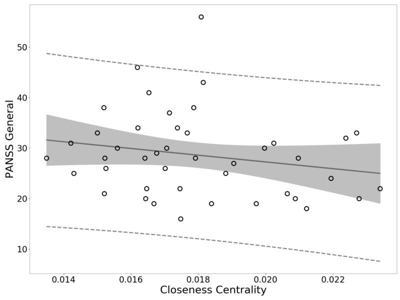

(d) $r = -0.205$, $p = 0.186$

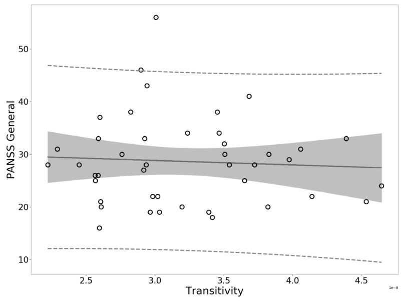

(e) $r = -0.062$, $p = 0.691$

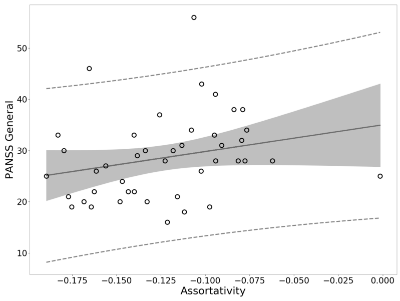

(f) $r = 0.247$, $p = 0.110$

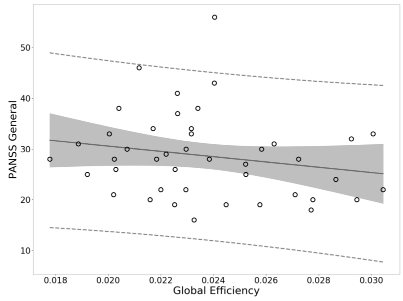

(g) $r = -0.201$, $p = 0.120$

Figure S7: Correlation of graph measures of the SCZ group with PANSS general scores. a) characteristic path length, b) cluster coefficient, c) betweenness centrality, d) closeness centrality, e) transitivity, f) assortativity, and g) global efficiency. The line corresponds to the fitted trend line and the shaded region the 95% confidence interval.

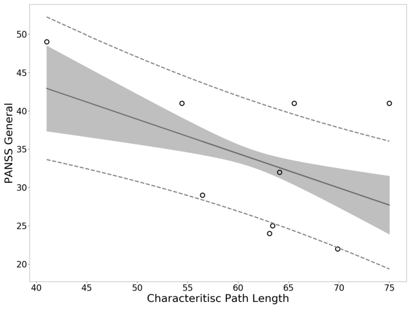

(a) $r = -0.463$, $p = 0.209$

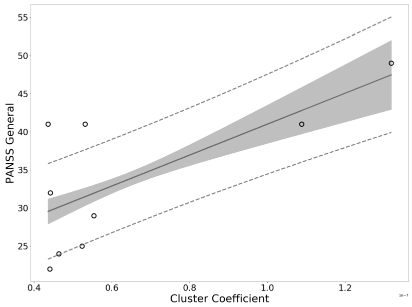

(b) $r = 0.693$, $p = 0.038$

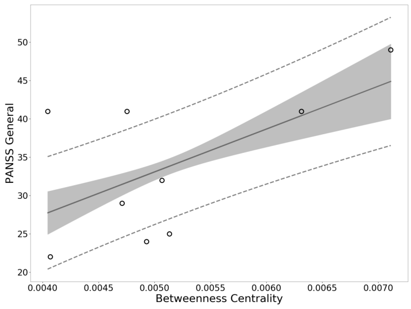

(c) $r = 0.587$, $p = 0.096$

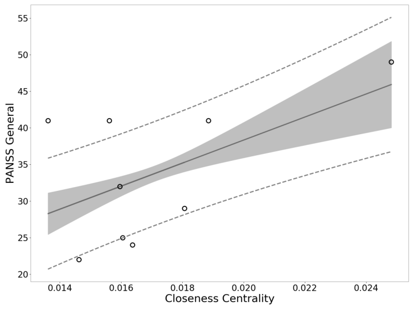

(d) $r = 0.546$, $p = 0.129$

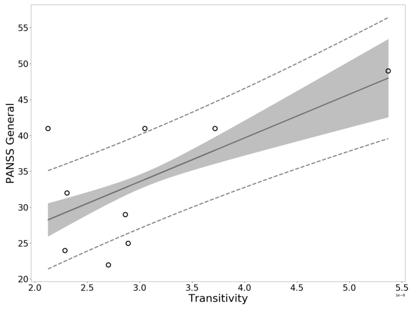

(e) $r = 0.639$, $p = 0.064$

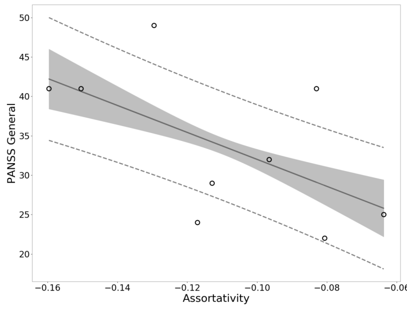

(f) $r = -0.584$, $p = 0.099$

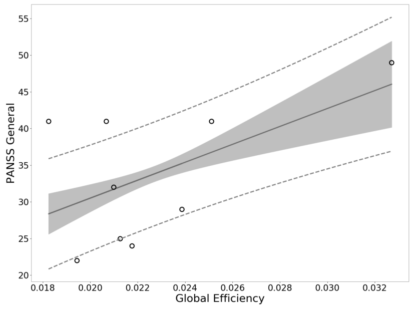

(g) $r = 0.552$, $p = 0.124$

Figure S8: Correlation of graph measures of the SCZaff group with PANSS general scores. a) characteristic path length, b) cluster coefficient, c) betweenness centrality, d) closeness centrality, e) transitivity, f) assortativity, and g) global efficiency. The line corresponds to the fitted trend line and the shaded region the 95% confidence interval.

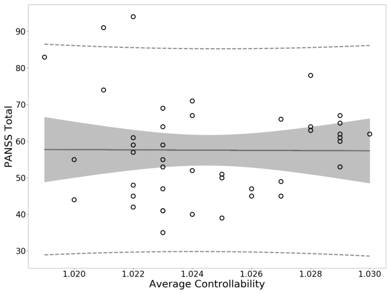

(a) $r = -0.001$, $p = 0.965$

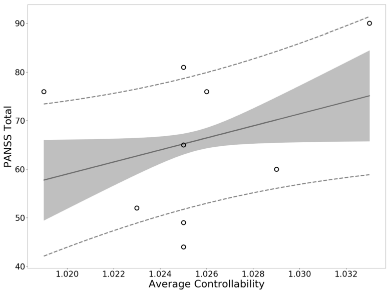

(b) $r = 0.301$, $p = 0.431$

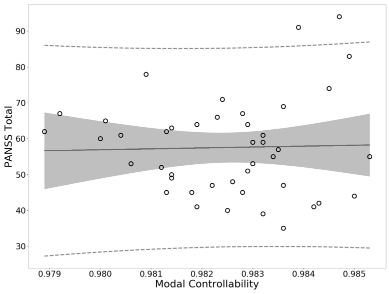

(c) $r = 0.029$, $p = 0.856$

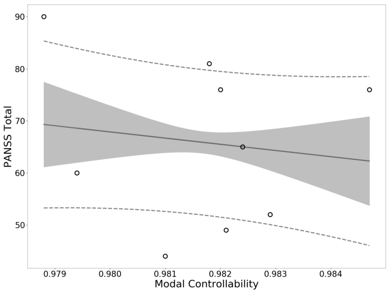

(d) $r = -0.134$, $p = 0.731$

Figure S9: Correlation of controllability measures with PANSS total scores. a) average controllability (SCZ), b) average controllability (SCZaff), c) modal controllability (SCZ), and d) modal controllability (SCZaff). The line corresponds to the fitted trend line and the shaded region the 95% confidence interval.

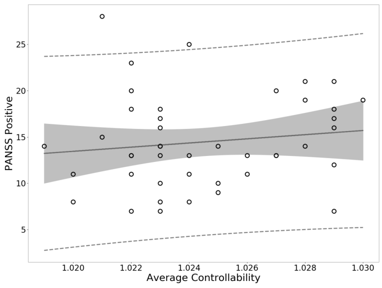

(a) $r = 0.134$,$p = 0.390$

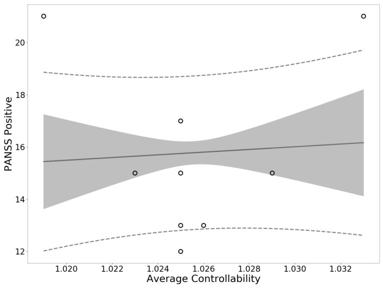

(b) $r = 0.060$,$p = 0.878$

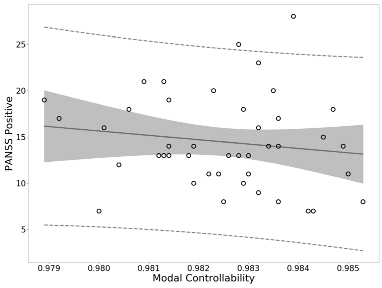

(c) $r = -0.146$,$p = 0.249$

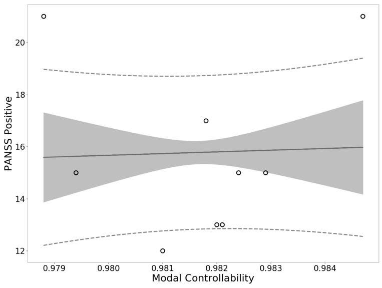

(d) $r = 0.035$,$p = 0.929$

Figure S10: Correlation of controllability measures with PANSS positive scores. a) average controllability (SCZ), b) average controllability (SCZaff), c) modal controllability (SCZ), and d) modal controllability (SCZaff). The line corresponds to the fitted trend line and the shaded region the 95% confidence interval.

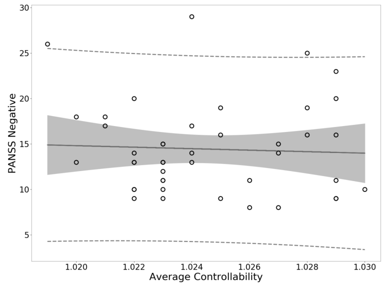

(a) $r = -0.049$,$p = 0.755$

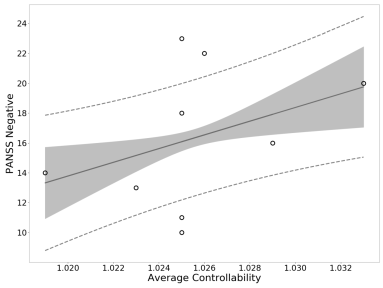

(b) $r = 0.375$,$p = 0.321$

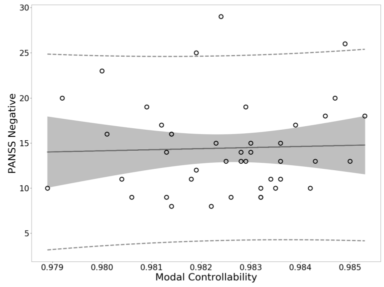

(c) $r = 0.037$,$p = 0.812$

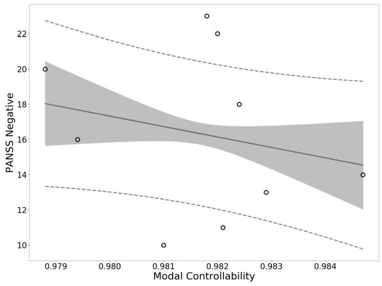

(d) $r = -0.224$,$p = 0.563$

Figure S11: Correlation of controllability measures with PANSS negative scores. a) average controllability (SCZ), b) average controllability (SCZaff), c) modal controllability (SCZ), and d) modal controllability (SCZaff). The line corresponds to the fitted trend line and the shaded region the 95% confidence interval.

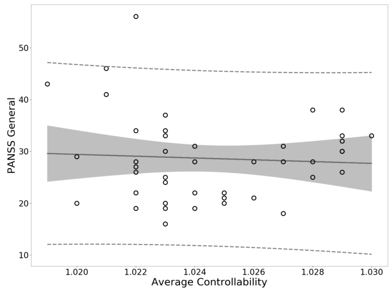

(a) $r = -0.062$,$p = 0.691$

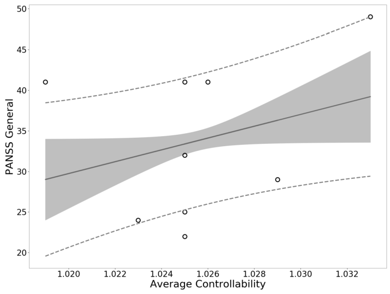

(b) $r = 0.294$,$p = 0.443$

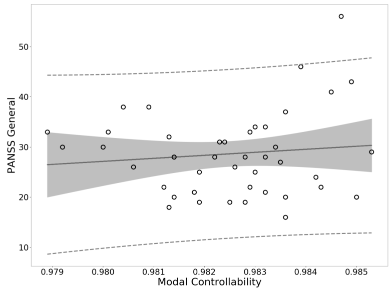

(c) $r = 0.112$,$p = 0.473$

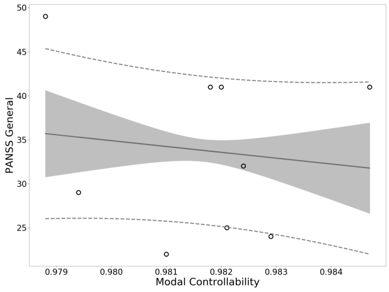

(d) $r = -0.124$,$p = 0.750$

Figure S12: Correlation of controllability measures with PANSS general scores. a) average controllability (SCZ), b) average controllability (SCZaff), c) modal controllability (SCZ), and d) modal controllability (SCZaff). The line corresponds to the fitted trend line and the shaded region the 95% confidence interval.

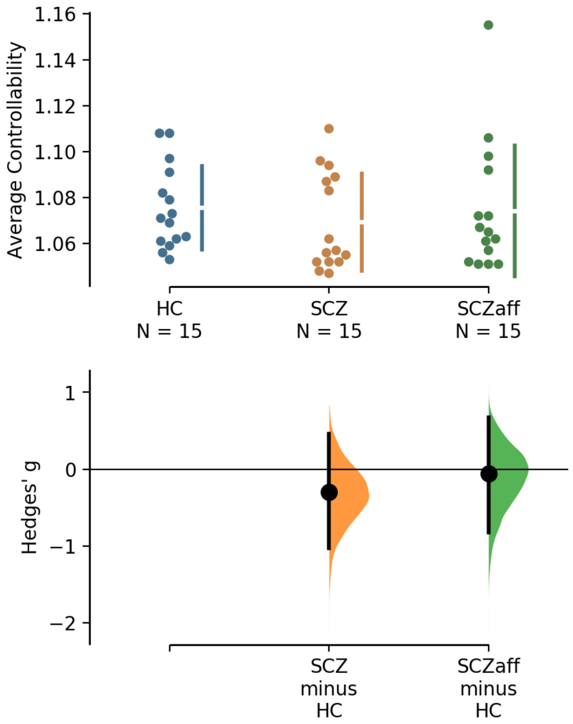

(a) Average controllability

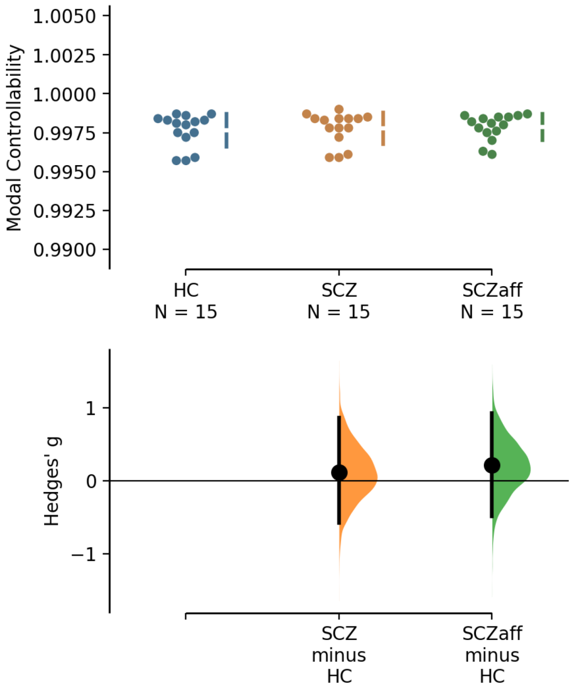

(b) Modal controllability

Figure S13: Cognitive systems and controllability. Hedge's g for the comparisons of (a) average and (b) modal controllability of weighted graphs of the SCZ and SCZaff groups against the HC group are shown in the above Cumming estimation plot. Here, we restricted the analysis to the top 15 regions with highest average and modal controllability (averaged over all subjects), respectively. See also Figure 4 in the main manuscript. The raw data is plotted on the upper axes. On the lower axes, Hedge's g is plotted as bootstrap sampling distributions. Each g is depicted as a dot. Each 95% confidence interval is indicated by the ends of the vertical error bars.

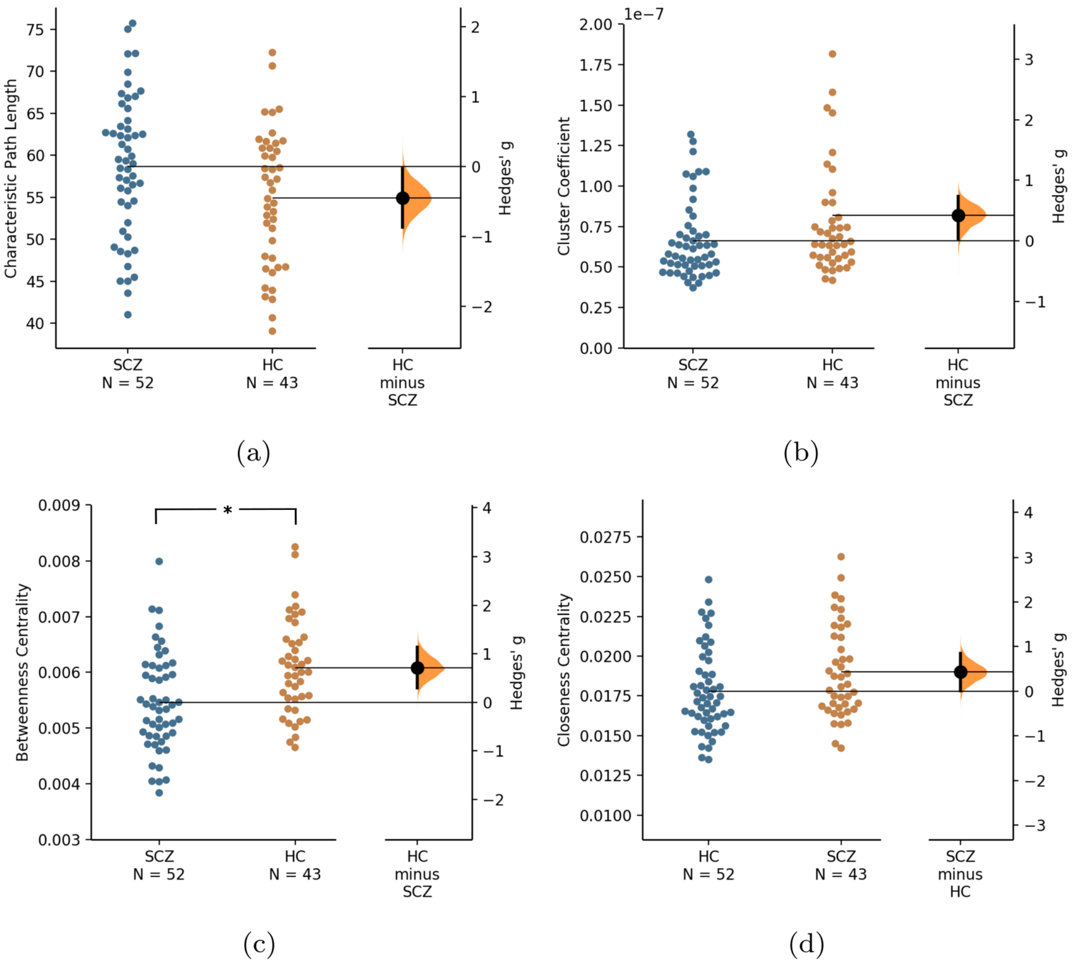

Figure S14: Hedge's g for the comparisons of various graph measures for the pooled SCZ and SCZaff groups (denoted by SCZ) against the HC group are shown in the above Cumming estimation plot. The raw data is plotted on the upper axes. On the lower axes, mean differences are plotted as bootstrap sampling distributions. Each mean difference is depicted as a dot. Each 95% confidence interval is indicated by the ends of the vertical error bars. Specifically, panels show a) characteristic path length (measure of integration) b) cluster coefficient (measure of segregation), c) betweenness centrality (measure of centrality) and d) closeness centrality (measure of centrality).* denotes statistically significant differences at a significance level of 0.05 Bonferroni corrected.

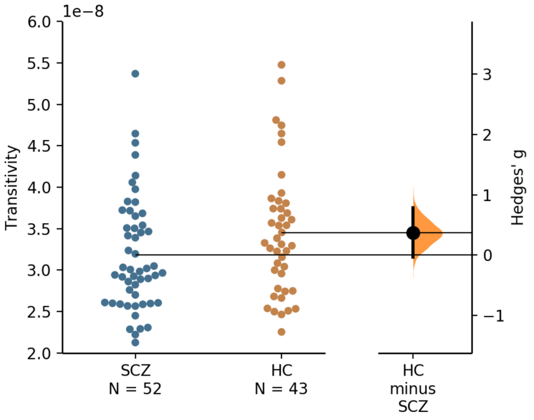

(a)

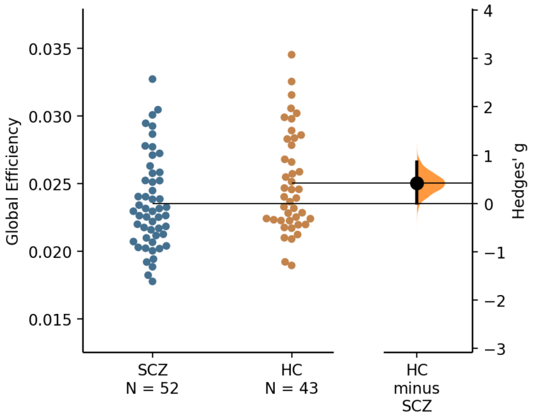

(b)

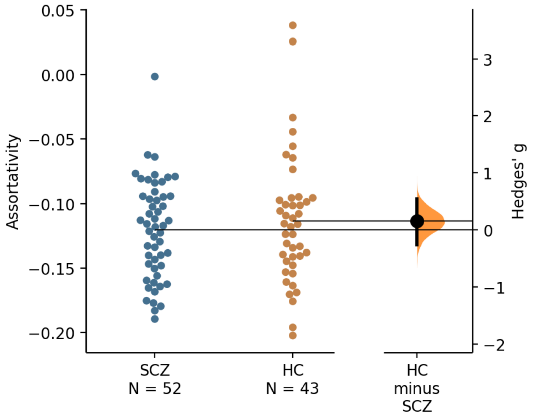

(c)

Figure S15: Hedge's g for the comparisons of various graph measures for the pooled SCZ and SCZaff groups (denoted by SCZ) against the HC group are shown in the above Cumming estimation plot. The raw data is plotted on the upper axes. On the lower axes, mean differences are plotted as bootstrap sampling distributions. Each mean difference is depicted as a dot. Each 95% confidence interval is indicated by the ends of the vertical error bars. Specifically, panels show a) transitivity (measure of segregation) b) global efficiency (measure of integration), and c) assortativity (measure of resilience).* denotes statistically significant differences at a significance level of 0.05 Bonferroni corrected.

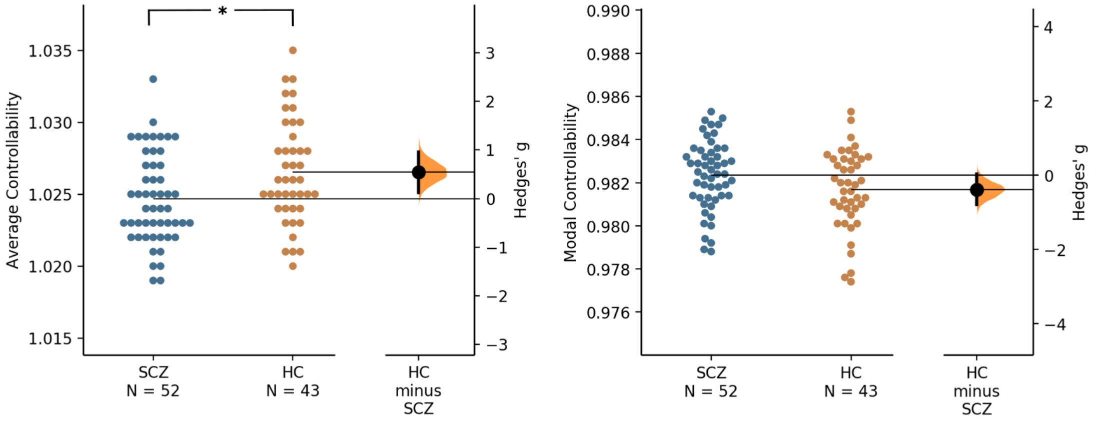

(a) Average Controllability (b) Modal controllability

Figure S16: Hedge's g for the comparisons of (a) average and (b) modal controllability of unweighted graphs of the pooled SCZ and SCZaff group (denoted by SCZ) against the HC group are shown in the above Cumming estimation plot. The raw data is plotted on the upper axes. On the lower axes, Hedge's g is plotted as bootstrap sampling distributions. Each g is depicted as a dot. Each 95% confidence interval is indicated by the ends of the vertical error bars.
